# ParAB Partition Dynamics in Firmicutes: Nucleoid Bound ParA Captures and Tethers ParB-Plasmid Complexes

**DOI:** 10.1371/journal.pone.0131943

**Published:** 2015-07-10

**Authors:** Virginia S. Lioy, Andrea Volante, Nora E. Soberón, Rudi Lurz, Silvia Ayora, Juan C. Alonso

**Affiliations:** 1 Department of Microbial Biotechnology, Centro Nacional de Biotecnología, CNB-CSIC, Darwin Str. 3, 28049 Madrid, Spain; 2 Max Planck Institute for Molecular Genetics, Ihnestrasse 73, D-1000 Berlin, Germany; University of Oklahoma, UNITED STATES

## Abstract

In Firmicutes, small homodimeric ParA-like (δ_2_) and ParB-like (ω_2_) proteins, in concert with cis-acting plasmid-borne *parS* and the host chromosome, secure stable plasmid inheritance in a growing bacterial population. This study shows that (ω:YFP)_2_ binding to *parS *facilitates plasmid clustering in the cytosol. (δ:GFP)_2_ requires ATP binding but not hydrolysis to localize onto the cell’s nucleoid as a fluorescent cloud. The interaction of (δ:CFP)_2_ or δ_2_ bound to the nucleoid with (ω:YFP)_2_ foci facilitates plasmid capture, from a very broad distribution, towards the nucleoid and plasmid pairing. *parS*-bound ω_2_ promotes redistribution of (δ:GFP)_2_, leading to the dynamic release of (δ:GFP)_2_ from the nucleoid, in a process favored by ATP hydrolysis and protein-protein interaction. (δD60A:GFP)_2_, which binds but cannot hydrolyze ATP, also forms unstable complexes on the nucleoid. In the presence of ω_2_, (δD60A:GFP)_2_ accumulates foci or patched structures on the nucleoid. We propose that (δ:GFP)_2_ binding to different nucleoid regions and to ω_2_-*parS* might generate (δ:GFP)_2_ gradients that could direct plasmid movement. The iterative pairing and unpairing cycles may tether plasmids equidistantly on the nucleoid to ensure faithful plasmid segregation by a mechanism compatible with the diffusion-ratchet mechanism as proposed from *in vitro* reconstituted systems.

## Introduction

In eukaryotes, much insight has been gained into how chromosomes are segregated. In contrast, much less is known in prokaryotes. The ParAB partition system, which is the only type present in bacteria, is the most widespread system among low-copy number plasmids. This system relies on four components: ParA and ParB proteins, cis-acting plasmid-borne *parS* DNA and the host genome [1,2]. In general the ParA and ParB proteins are subdivided in two subfamilies based on their size [3]. The large ParA ATPases (e.g., P1-ParA or F-SopA) contain two DNA binding domains: an N-terminal sequence-specific and a C-terminal non-specific (ns) DNA binding domain [4–6]. The small ParA ATPases, which lack the N-terminal sequence-specific DNA binding motif, bind nsDNA by forming either filaments (e.g., pB171-ParA) [7], discrete blobs (e.g., pSM19035-δ_2_) [8], or they might form bundles (e.g., TP228-ParF) in the absence of any support [9].

The ParB centromere binding proteins (CBPs) are subdivided also into two structurally unrelated groups. The first group includes large (or medium size) dimeric helix-turn-helix proteins (e.g., P1-ParB, F-SopB and chromosomal-encoded ParB) that bind to *parS* and to nsDNA to form large nucleoprotein complexes [10–14]. These CBPs, upon binding to *parS*, co-operatively spread over nsDNA many kilobases (kb) and promote bridging, looping and condensation of nsDNA [10–12,15]. The second group includes small dimeric ribbon-helix-helix ParB proteins (e.g., pSM19035-ω_2_, TP228-ParG, pB171-ParB). pSM19035-ω_2_ specifically binds to *parS* to form ordered helical structures without significant spreading into nsDNA [8,16,17].

**Fig 1:**
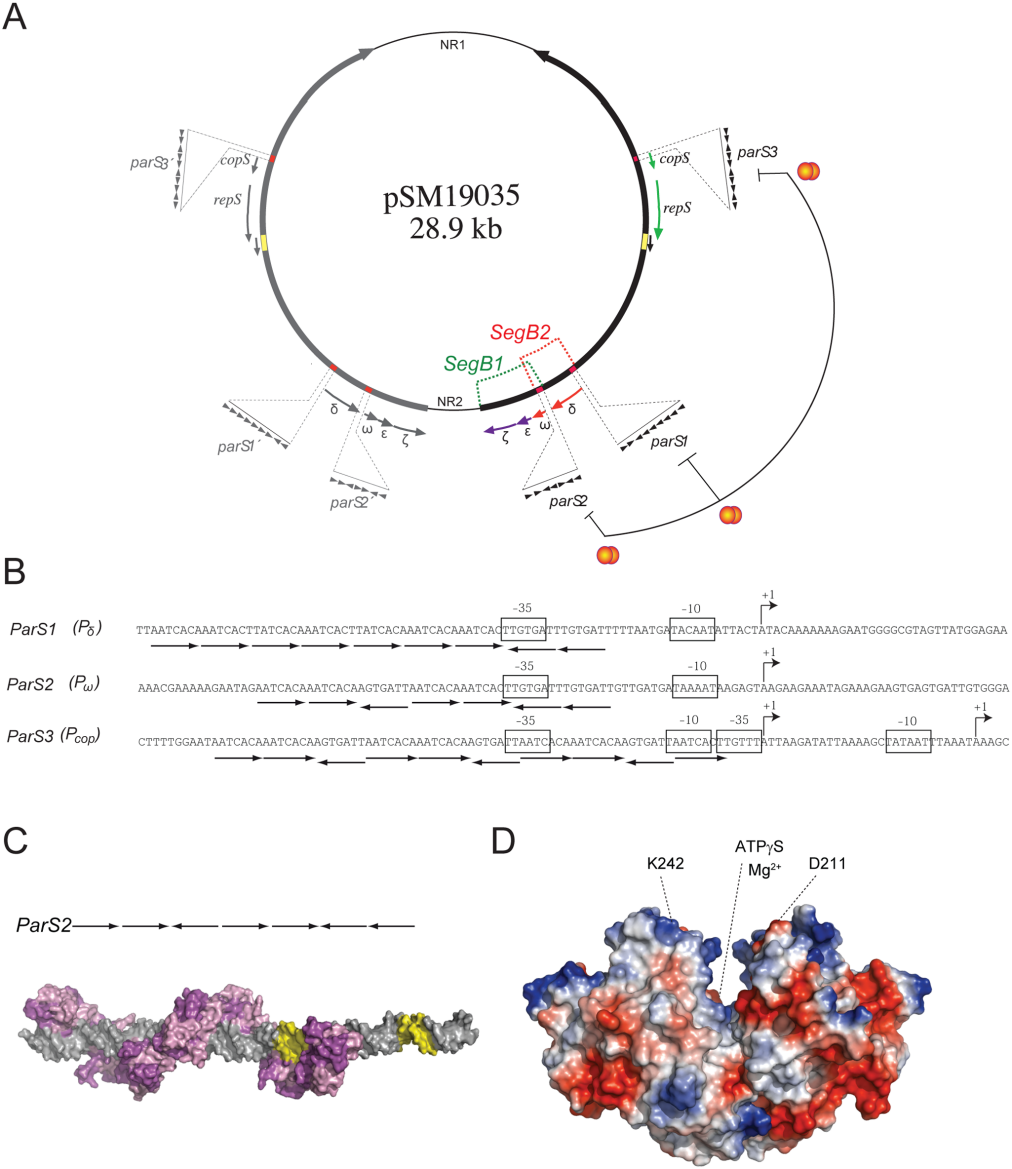
Genome organization, δ_2_ structure and proposed ω_2_-*parS* complex. (A) Plasmid pSM19035 map indicating the duplicated (thick arrows) and the unique non-repeated sequences (thin lines). The replication origin (yellow box) and direction of replication (denoted by arrows) are indicated. The upstream region of the promoters of the *copS*, δ and ω genes (red boxes), which constitute the six *parS* sites, are enlarged. The variable number of 7-bp repeats (iterons) is symbolized by filled arrowheads (▶ or ◀). The promoters repressed by ω_2_ (red balls) are indicated. The SegB1 (ω_2_, ε_2_ and ζ) and the SegB2 (δ_2_, ω_2_) loci are indicated. For simplicity all these features are colored and highlighted in the duplicated region located at the right of the plasmid, but the same applies for the other long inverted repeat. (B) The *parS* sites consist of a variable number of contiguous iterons present in three different promoter regions. The boxes denote the -35 and -10 consensus sequences and the bent arrows denote the +1 position of the transcripts. (C) Model of seven ω_2_ bound to *parS*2 DNA based on the crystal structures determined for [ω_2_ΔN19]_2_-(→→) and [ω_2_ΔN19]_2_-(→←) complexes (PDB 1IRQ, 2BNW, 2BNZ and 2CAX) [16]. DNA is shown in grey with the -35 and -10 sequences in yellow, and ω_2_ in surface representation (one monomer is purple, the other violet). (D) Electrostatic potential surface representation of δ_2_ in the ATPγS·Mg2^+^ bound form (PDB 2OZE) displayed using PyMOL. The surface charge of δ_2_ is negative (red) near the bottom of the U, and positive (blue) at the tips of the arms of the U. The relevant region involved in nsDNA binding maps at the tips of the arms of its U-shaped structure. The localization of two residues involved in nsDNA binding used in this work are indicated by dotted lines (each one located in one monomer).

The interaction of the ParA and ParB components, which leads to proper separation of plasmid copies, has been extensively studied in plasmids and bacteria of the Proteobacteria phylum. These studies provide the foundation for filament- and non-filament-based modes of plasmid and bacterial chromosome segregation. In the filament-based modes, small ParA, when bound to ATP (ParA-ATP), assembles into bundles, and the partition complexes are mobilized by linear contractile filaments in a manner reminiscent of the spindle mechanism in eukaryotes (thread pushing or pulling model) [9,18]. Alternatively, ParA assembles by forming nucleoprotein filaments, and the partition complexes are mobilized by contractile helical or linear filaments as a cargo (filament-pulling model) [7,19]. In the non-filament-based mode (diffusion-ratchet and DNA relay models), small or large ParA-ATP binds to the nucleoid as dimers or small oligomers [8,20–25]. In the diffusion-ratchet model, a propagating large ParA ATPase gradient is the driving force for movement of the partition complexes [22–24], whereas in the DNA relay model, the forces that drive segregation are generated by the small ParA gradient and the elastic forces within the DNA molecule [25]. Very little is known about the mechanisms that lead to accurate segregation of small ParA- and ParB-like proteins in plasmids of the Firmicutes phylum. It was previously shown that the almost absolute segregational stability of plasmids belonging to the *inc18* family requires at least two active stabilization systems, the partition (ParAB or SegB2) and toxin-antitoxin (SegB1) systems (Fig 1A). Plasmids of the *inc18* family (pSM19035 being its representative) require homodimeric small ParA-like δ (δ_2_) and small ParB-like ω (ω_2_) products as well as *parS* to ensure faithful segregation (Fig 1B) [26]. In pSM19035, the *parS* sites, which comprise 7 to 10 contiguous heptads, overlap with the promoter (*P*) regions of the δ (*P*_δ_ or *parS*1), ω (*P*_ω_ or *parS*2) and *cop* (*P*_*cop*_ or *parS*3) genes (Fig 1B) [8,16,17,27]. Faithful segregation of a plasmid bearing the ω gene, transcribed from its own promoter *P*_ω_, is not significantly impaired in comparison with its natural context if the expression of the (δ:*gfp*) gene (integrated into the bacterial chromosome and transcribed from an IPTG-inducible promoter, *P*_hsp_) mimics its native concentration [28]. It is likely therefore that a single *parS* site may be sufficient for stable plasmid segregation, but in its natural context *parS*1, *parS*2 and *parS*3 are present (Fig 1A).

The structure of CBP ω_2_ or its variant ω_2_ΔN19, which lacks the first 19 amino acids, bound to minimal sub-sites allowed us to understand how ω_2_ binds to *parS* DNA (Fig 1C) [16,29]. *In vitro*, ω_2_ or ω_2_ΔN19 transiently binds with high affinity and co-operativity to *parS* DNA (apparent dissociation constant [K_Dapp_] 5 ± 1 nM) [30–32]. The interaction of the unstructured N-terminal end of ω_2_ with δ_2_ (even in the apo form) increased the binding affinity of ω_2_ for *parS* DNA ∼8-fold (K_Dapp_ 0.7 ± 0.1 nM) and the half-life of the ω_2_-*parS* DNA complex >20-fold [17].

Protein δ_2_ has a U-shaped structure, with each of the arms and the joining region representing one monomer (Fig 1D). The C-terminal nsDNA binding domain lies at the tip of the arms of the U structure (highlighted by the D211 and K242 residues in Fig 1D) [27]. *In vitro*, wild type [wt] δ_2_ bound to ATP·Mg^2+^ (denoted as ATP) binds to nsDNA, forming discrete complexes. These complexes, which show spherical or blob shapes rather than a nucleoprotein filament, contain up to 5 ± 1 δ_2_/blob as shown by atomic force microscopy (AFM) [8]. In the absence of nsDNA, however, wt δ_2_-ATP free in solution forms discrete blob shaped structures containing 2–3 δ_2_/blob, rather than long bundles [8]. The interaction of δ_2_·ATP bound to nsDNA with wt ω_2_ bound to *parS* facilitates plasmid-nucleoid pairing *in vitro* [8,27]. Biochemical analysis also showed that stoichiometric ω_2_ concentrations stimulate the ATPase activity of δ_2_, resulting in dissociation of δ_2_ from nsDNA and plasmid-nsDNA unpairing [17,27].

We report here that *in vivo* (ω:YFP)_2_ binding to a plasmid-borne *parS* site causes discrete clustering of plasmid copies and that (δ:GFP)_2_ bound to the *Bacillus subtilis* genome forms dynamic clouds over the nucleoid. The interaction of (δ:GFP)_2_ bound to the nucleoid with wt ω_2_ or (ω:YFP)_2_ bound to *parS* captures and tethers plasmids at the nucleoid, as reported from *in vitro* analyses [8,27]. Then, the ω_2_-*parS* complex stimulates the δ_2_ or (δ:GFP)_2_ ATPase activity, and ATP hydrolysis facilitates the disassembly of δ_2_. The iterative assembly/disassembly cycles may transduce the chemical energy produced by the motor protein into unidirectional plasmid movement.

## Materials and Methods

### Strains and plasmids

The *B*. *subtilis* strains used are listed in S1 Table. In BG1311, the 3´-end of the *lacI* gene was fused to *gfp* gene, to render the *lacI*:*gfp* gene that was placed under the control of the xylose-inducible promoter, and integrated, by a double crossover event, as a unique sequence at the *amy* locus of BG214 cells. In BG1469 and BG1447, the promoter-less ω:*yfp* and ωΔN19:*yfp* genes were placed under the transcriptional control of the IPTG-inducible *P*_*hsp*_ promoter, and integrated, by a double crossover event, as unique copy at the *amy* locus of BG214 cells. The plasmids used for localization studies, based in the pHP14 vector, were grown in *B*. *subtilis* and are listed in S1 Table. The δ gene encodes two co-linear polypeptides, a 298-residies (δ_+14_) and a 284-residues product. The structure of δ_+14_ (having 14 extra N-terminal residues) bound to ATPγS and Mg^2+^ includes all 284 residues of the wt δ protein [27]. The plasmid-based wt δ gene and its variants were under the control of its own promoter (*P*_δ_), which overlaps with the *parS*1 site, and the ω gene and its variants were under the controls of its own promoter (*P*_ω_), which overlaps with *parS*2 (S1 Table, Fig 1B). The plasmids used for overexpression, based in the pT712 vector, were grown in *E*. *coli* ER2566 (Biolabs), and are listed in S1 Table.

### Plasmid copy number, plasmid stability test, β-galactosidase assays and *in vivo* ω_2_ and δ_2_ concentrations

The number of plasmid copies per cell was estimated by hybridization and by quantitative PCR and normalization with two distinct chromosomal genes as previously described [21,33]. To determine the frequency of plasmid loss, cells bearing plasmids were grown for more than 100 generations in S7 minimal medium (MMS7). After 8 h incubation at 30°C (~12 generations), a fraction of the culture was diluted into pre-warmed fresh antibiotic-free MMS7 medium, and grown again for 8 h at 30°C. This dilution and growth was repeated until the 100 generations were reached. The number of cells containing plasmid (conferring chloramphenicol resistance) was determined at different time intervals by plating appropriate dilutions in LB plates and then replica plating onto chloramphenicol-containing plates. The relative loss rate is expressed as a percentage and calculated as L = (L_N_-L_X_)/(L_N_-L_P_) X 100, where L_N_ is the loss rate per cell generation of negative control (empty vector), L_X_ is the empirical loss rate of vector-bearing δ_2_ and ω_2_ variants, and L_P_ is loss rate per cell generation of positive control (vector-bearing δ and ω wt genes).

The promoter-less *lac*Z read from the *P*_δ_ promoter (*P*_δ_ *lac*Z fusions), integrated into the *amyE* locus (BG508 strain), was used for *in vivo* transcription experiments. β-galactosidase assays were performed as described [30] except that the centrifuged *B*. *subtilis* cells were resuspended and lysed by the addition of 0.1% sodium dodecyl sulfate (SDS) (final concentration 0.0025%) and chloroform (final concentration 2%). The activity is expressed in Miller units after small modifications as described [30].

To quantify protein levels, *B*. *subtilis* cells bearing plasmid-borne δ, ω, δ and ω gene(s) (or their mutant variants), under their native (or IPTG-induced) control (see S1 Table), were grown in LB to an OD_560_ = 0.4 at 37°C with agitation in the presence of chloramphenicol (5 μg/ml). The cells were harvested, resuspended in buffer A (50 mM Tris HCl [pH 7.5], 300 mM NaCl, 5% glycerol) and lysed by sonication. Extracts containing equal concentrations of protein from each condition alongside purified ω and δ protein standards (5 to 500 ng) were separated by 15% SDS-polyacrylamide gel electrophoresis (SDS-PAGE). Mouse polyclonal anti-δ_2_ and rabbit polyclonal anti-ω_2_ antibodies were obtained using standard techniques [27]. Immunoblots were transferred and probed with the antibodies as described previously [34]. Protein ω and δ bands, on developed immunoblots, were quantified with a scanning densitometer (Quantity One software, BioRad). Purified ω and δ protein standards yielded a linear relationship between antibody signal and the protein concentration. The amount of ω and δ protein in each sample was interpolated from the standard curve obtained with purified protein, and the *in vivo* concentration of ω and δ was estimated considering a cell volume of 1.2 femtoliters and based on 5 x 10^7^ colony-forming unit (CFU)/ ml at an OD_560_ of 0.4. Since >95% of BG214 cells were singlets and doublets, a correlation of CFU per cell averaged to 1.6.

### Chemicals, enzymes, proteins, DNA and reagents

All chemicals were p.a. grade and purchased from Roche Diagnostics (Mannheim, Germany). DNA restriction, DNA modification enzymes and nucleotides were from New England Biolabs and Sigma. Ultrapure acrylamide was from Serva (Heidelberg, Germany). The broad protein molecular weight marker was obtained from GIBCO-BRL (Barcelona, Spain). Proteins ω_2_, δ_2_, δ_2_D60A, δ_2_D211A, δ_2_D60A δ_2_D211A, and δ_2_K242A and pBC30-borne *parS*2 DNA, which is the source of *parS* DNA, were purified as described [27,30,32].

The concentration of DNA was expressed as moles of DNA molecules and was determined using a molar extinction coefficient of 6,500 M^-1^cm^-1^ at 260 nm. The protein concentrations were determined by absorption at 280 nm using molar extinction coefficients of 2,980 M^-1^ cm^-1^ for ω_2_, and 38,850 M^-1^ cm^-1^ for δ_2_, δ_2_D60A, δ_2_D211A, δ_2_D60A δ_2_D211A and δ_2_K242A. Concentrations are expressed as moles of protein dimers. It must be noted that unless stated otherwise, δ_2_ or its mutant variants in the ATP bound form are denoted as δ_2_, in the presence of ADP-Mg^2+^ as δ_2_-ADP, and in the absence of a nucleotide co-factor as apo-δ_2_, respectively.

### Protein-DNA complexes

For electrophoretic mobility shift assays (EMSA), gel-purified 423-bp [α ^32^P]-*Hin*dIII-*Kpn*I *parS*2 DNA (0.1 nM) was incubated with various amounts of wt ω_2_, wt δ_2_ (or its variants), or both proteins in buffer B (50 mM Tris-HCl [pH 7.5], 50 mM NaCl, 10 mM MgCl_2_) containing or lacking 1 mM ATP or ADP for 15 min at 37°C in 20 μl final volume. The reactions were stopped by addition of loading buffer (1 mM EDTA, 0.1% [v/v] bromophenol blue, and 0.1% [v/v] xylene cyanol) and were then separated using 4 or 6% PAGE. PAGE was conducted in 1x TAE running buffer at 200 V at 4°C, and the gels were dried prior to autoradiography.

To obtain apparent dissociation constant (K_Dapp_) values from EMSA experiments, the concentrations of free DNA and protein-DNA complexes were densitometrically determined from differently exposed autoradiographs of EMSA gels. Protein concentrations that transfer 50% of the labeled DNA into complexes are approximately equal to the K_Dapp_ under conditions where the DNA concentration is much lower than the K_Dapp_.

The structural images were generated using PyMOL Molecular Graphics System, Version 1.5.0.4 (Schrödinger, LLC).

### Fluorescence and electron microscopy

*B*. *subtilis* cells bearing the indicated plasmid or expression cassette were grown overnight in MMS7 medium, in the presence of chloramphenicol or spectinomicin, at 30°C. The cultures were diluted in fresh medium to OD_560_ ~0.05 and incubated until OD_560_ ~0.4. Synthesis of the LacI:GFP fusion, from the BG1311strain, was induced by addition of xylose (0.5%). Plasmid-borne ω:*yfp*, δ:*gfp* or δD60A:*gfp* genes were expressed from their native promoters. When indicated IPTG (10 μM final concentration) was added to BG947 or BG1097 cells to induce synthesis of chromosomal-encoded δ:*gfp* or δD60A:*gfp* gene. In the absence of IPTG, cellular autofluorescence was not observed. For nucleoid visualization, the sample (1.5 ml) was incubated with DAPI (final concentration 5 μg/ml) on ice and in darkness for 10 min before slide preparation [27]. The cells were harvested (1.5 ml), centrifuged, and the pellet resuspended in pre-warmed MMS7 medium (50 μl). An aliquot was placed on a polylysine-coated glass slide and covered with a coverslip, and incubated at 30°C as previously described [21]. Images were acquired using a Nikon Eclipse E-1000 fluorescence microscope equipped with a Nikon C-CU Universal condenser, a Smrock GFP-3035 bright-line zero band-pass filter cube, and a Hamamatsu Orca-ER c4742-95 charge-couple device (CCD) camera. Time-lapse photo-microscopy, with images gathered every 20 s over a 10 min period, was carried out with cells growing as micro-colonies on a slide, and analyzed with the Image Pro Plus 6.1 software using macro-directed cell recognition and measurement of the focus number and position as described [21].

Circular pCB30 harboring *parS*2 DNA (5 nM) was incubated with the indicated protein(s) for 15 min at 37°C in buffer C (50 mM TEA [pH 7.5], 50 mM NaCl, 10 mM MgCl_2_, 1 mM ATP) as previously described [31]. After negative staining with 1% uranyl acetate or after fixation with 0.2% (v/v) glutaraldehyde for 10 min at room temperature, the DNA-protein complexes were visualized by electron microscopy (EM) [27,35]. The procedures for adsorption of the complexes to mica, rotational shadowing with platinum, and EM image evaluation have been previously described [27].

## Results

### Contribution of δ_2_ and ω_2_ to segregation stability

**Fig 2:**
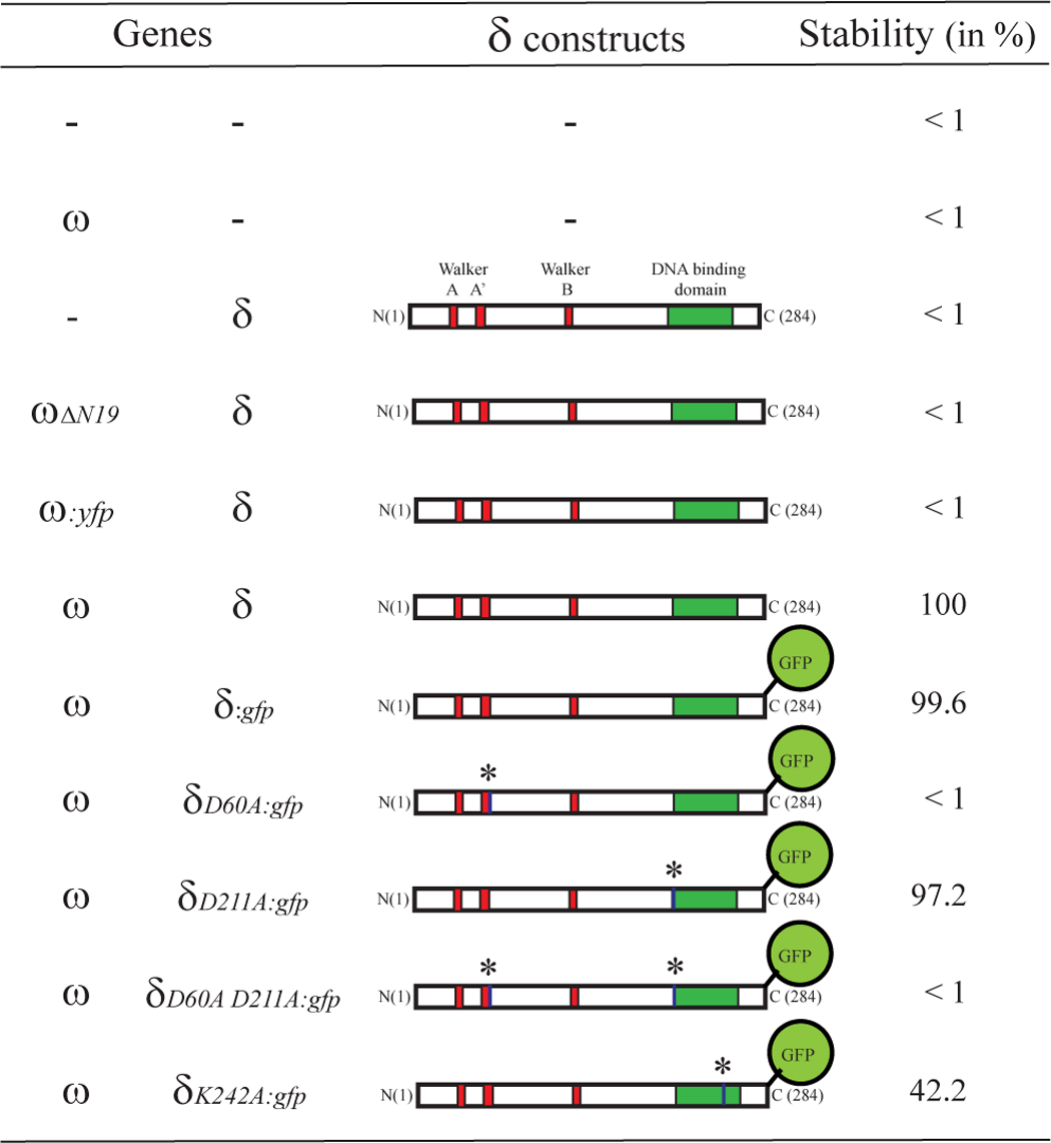
Scheme of the different ω_2_ and δ_2_ variants used and their contribution to plasmid stability. Essential domains in the δ_2_ protein are highlighted and the asterisks indicate the position of the mutated residues. The Par^-^ pHP14 vector and derivatives (~8 copies per cell) bearing the whole pSM19035 *par* locus or part of it were grown in LB medium at 30°C for at least 120 generations of growth, and plasmid stability was measured as described in the Materials and Methods section.

The functionality of the proteins analyzed in this work was tested using the plasmid stabilization assay described previously [27]. The δ and ω gene products and *parS*1 (*P*_δ_) and *parS*2 (*P*_ω_) are necessary to stabilize an unstable and unrelated plasmid replicon (Fig 2, S1 Table). However, under certain conditions (i.e., when the δ gene is transcribed from a IPTG inducible promoter (*P*_hsp_), see below) a single *parS* site is sufficient to stabilize plasmid segregation [28].

When δ_2_ was replaced by fused δ:*gfp*, which is also a dimer in solution (δ:GFP)_2_, faithful plasmid segregation was observed (Fig 2). Similar results were observed when δ was replaced by the fused δ:*cfp* gene (data not shown). Previously it was shown that: i) ATP-bound δ_2_ binds nsDNA, but ADP-bound δ_2_ and apo-δ_2_ do not bind nsDNA (S2 Table) and ii) ATP-bound δ_2_D60A, which is unable to hydrolyze ATP, binds nsDNA with a 2-fold higher affinity (S2 Table) [27]. When δ_2_ was replaced by (δD60A:GFP)_2_ plasmids were randomly segregated (Fig 2). These results suggest that the C-terminal fusion does not affect the activity of δ_2_, and that ATP hydrolysis is essential for plasmid segregation.

To gain insight into how δ_2_ binding affinity to nsDNA contributes to plasmid segregation a screening assay was performed. An exchange of a single negatively charged residue (e.g., δ_2_D211) to alanine in the DNA binding domain resulted in *in vitro* binding to nsDNA with > 6-fold higher affinity than wt δ_2_ (S2 Table). When δ_2_ was replaced by the δ_2_D211A variant plasmids were faithfully segregated (Fig 2). However, in the absence of ATP hydrolysis, increased affinity for nsDNA (i.e., in the δ_2_D60A D211A variant, S2 Table) was not sufficient to facilitate faithful plasmid segregation (Fig 2), suggesting that ATP hydrolysis rather than increased affinity for nsDNA is required for plasmid segregation.

Previous results showed that wt ω_2_ or ω_2_ΔN19 binds and represses *P*_δ_ utilization both *in vivo* and *in vitro* (S3 Table) [30], but the latter lacks the region essential for ω_2_-δ_2_ interaction [32]. When ω_2_ was replaced by ω_2_ΔN19 plasmid partitioning was impaired (Fig 2), suggesting that ω_2_-δ_2_ interaction is necessary for faithful plasmid segregation.

The ω gene was fused to the *yfp* gene at either the 5’- or 3’-end leading to *yfp*:ω and ω:*yfp* genes, respectively. When the ω gene was replaced by the *yfp*:ω or the ω:*yfp* gene, transcribed from its native *P*_ω_ (*parS*2), plasmid partition was impaired (Fig 2 and data not shown). *In vivo* experiments revealed that ω:YFP repressed *P*_δ_ utilization with an efficiency comparable to that of wt ω_2_. In the presence of δ_2_, ω:YFP further repressed *P*_δ_ utilization (S3 Table). Similar results were observed when ω:YFP was replaced by ω_2_, but not when ω_2_ΔN19, which fails to interact with the δ_2_, was used. It is likely therefore that ω:YFP interacts with δ_2_. Conversely, YFP:ω did not repress transcription from *P*_δ_, and the presence of δ_2_ did not overcome such defect (S3 Table). It is likely that: i) ω:YFP is a dimer in solution [i.e, further denoted as (ω:YFP)_2_] because only the dimeric form of the ribbon-helix-helix ω protein binds *parS* DNA [29,31]; ii) (ω:YFP)_2_ binds *parS*1 DNA and represses *P*_δ_ utilization, and interacts with δ_2_ as wt ω_2_, but (ω:YFP)_2_ bound to *parS* DNA fails to stimulate the δ_2_ ATPase activity (data not shown); and iii) the (ω:YFP)_2_-*parS* interaction can be used as a marker to localize plasmid DNA and for δ_2_-ω_2_ interaction. Similar results were reported for GFP:ParB of P1 plasmid, which was also used as marker of *in vivo* plasmid location. P1-GFP:ParB fails to promote proper plasmid segregation, but in the presence of GFP:ParB, a plasmid bearing P1-ParB and P1-ParA is accurately segregated [20,21]. P1-ParB does not contribute to the regulation of the *parAB* operon, and the cognate sequence of the promoter that transcribes both *parA* and *parB* genes is different from the *parS* sequence [3]. In contrast, in pSM10935 the *parS* sites overlap with *P*_δ_ and *P*_ω_, and ω_2_-mediated repression of both promoters leads to plasmid incompatibility [30,33]. To discriminate whether ω_2_ stimulation of the δ_2_ ATPase is essential for faithful partitioning or (ω:YFP)_2_ is a dominant negative variant, the stability of pBC706 (plasmid-borne *P*_ω_ ω and *P*_δ_ δ genes) was studied (S4 Table). Plasmid pCB706 was introduced into *B*. *subtilis* BG1469 or BG1447 cells bearing the ω:*yfp* or the ωΔN19:yfp gene, respectively, integrated as a unique sequence at the host *amyE* locus of the *B*. *subtilis* genome, transcribed from the IPTG-inducible promoter (*P*_*hsp*_) (S1 Table). In parallel, as a control, we introduced pBC706 or pCB586 (plasmid-borne *P*_ω_ ω gene) into the BG947 strain bearing *P*_*hsp*_ δ:*gfp*, integrated as a unique copy at the same locus (S1 Table). Since transcription of the three genes (ω:*yfp*, ωΔN19:*yfp* or δ:*gfp*) was driven by the same promoter (*P*_*hsp*_), and they are integrated into the same locus of the *B*. *subtilis* genome, it was expected that they would be expressed to the same degree.

At low transcription levels (10 μM IPTG), there were ~400 (ω:YFP)_2_/CFU (Table 1). Under this experimental condition, expression of (ω:YFP)_2_ partially reduced, by about 2-fold (47 ± 4%), pCB706 faithful partitioning (S4 Table) when compared to the absence of the *P*_*hsp*_ ω:*yfp* gene (BG214 cells bearing pCB706, Fig 2) or when IPTG was omitted (data not shown). Similar results were observed when *P*_*hsp*_ ω:*yfp* was replaced by BG1447-borne *P*_*hsp*_ ωΔN19:*yfp* bearing pBC706. Here, faithful pBC706 partitioning was also reduced about 2-fold (52 ± 3%) (S4 Table). However, in the presence of 50 μM IPTG, plasmid partitioning was impaired (< 1% of cells retained the plasmid after 100 generations) by the expression of the *P*_*hsp*_ ω:*yfp* or the *P*_*hsp*_ ωΔN19:*yfp* gene *in trans*. As previously shown [27], at low transcription levels (10 μM IPTG), expression of (δ:GFP)_2_ did not affect faithful pBC706 segregation, and it enhanced the segregation of pCB586 (plasmid-borne *P*_ω_ ω gene). However, the presence of 50 μM IPTG decreased the efficiency of pBC706 stable inheritance (S4 Table). It is likely that: i) (ω:YFP)_2_ is not dominant-negative over ω_2_; and ii) plasmid incompatibility and/or increased (ω:YFP)_2_- or (ωΔN19:YFP)_2_-mediated repression of the ω and δ genes might account for the decreased efficiency of plasmid inheritance.

**Table 1:** Protein (ω:YFP)_2_ binds *P*_δ_ and represses transcription.

Gene(s)^a^	Molecules/CFU^b^		Molar	
	(molecules/cell)		concentration/cell	
	Protein ω_2_	Protein δ_2_	Protein ω_2_	Protein δ_2_
*P*_ω_ ω (pCB586)	~1,300 (810)	NA	~1.1 μM	NA
*P*_ω_ ωΔN19 (pCB742)	~1,400 (875)	NA	~1.9 μM	NA
*P*_ω_ ω:*yfp* (pCB846)	~1,280 (800)	NA	~1.0 μM	NA
*P*_δ_ δ:*gfp* (pCB578)	NA	~7,500 (4,600)	NA	~6.0 μM
*P*_δ_ δD60A:*gfp* (pCB760)	NA	~7,700 (4,800)	NA	~6.2 μM
*P*_ω_ ω *P*_δ_ δ (pCB706)	~1,300 (810)	~1,400 (875)	~1.1 μM	~1.2 μM
*P*_ω_ ω:*yfp P*_δ_ δ (pCB843)	~1,320 (825)	~1,350 (850)	~1.1 μM	~1.1 μM
*P*_ω_ ω *P*_δ_ δD60A (pCB761)	~1,180 (810)	~1,380 (870)	~1.1 μM	~1.2 μM
*P_hsp_* δ:*gfp* (BG947)^c^	-	~6,500 (4,000)	-	~5.6 μM
*P_hsp_* δ:*gfp P*_ω_ ω (BG947)^c^ (pCB586)	~1,200 (750)	~6,600 (4,100)	~1.0 μM	~5.7 μM
*P_hsp_* δD60A:*gfp* (BG1097)^c^ (pCB586)	~1,300 (815)	~6,500 (4,000)	~1.1 μM	~5.6 μM
*P_hsp_* ω:*yfp* (BG1469)^c^	~400 (250)	NA	~0.3 μM	NA

^a^The plasmid or the strain bearing the relevant promoter(s) and gene(s) are indicated between parentheses.

^b^The molecules/CFU were estimated as described in Materials and methods. The estimated numbers of molecules/cell are denoted between parentheses.

^c^The *P*_*hsp*_ δ:*gfp*, *P*_*hsp*_ δD60A:*gfp* and *P*_*hsp*_ ω:*yfp* genes integrated as unique copies into the *amy* locus in *B*. *subtilis* are under the control of the LacI expression cassette (LacI repressor-Hyper-Spank promoter, *P*_*hsp*_) (see S1 Table). The BG947, BG1097 and BG1469 strains were grown in the presence of 10 μM IPTG. NA, not applicable.

### Experimental setup used to study plasmid partitioning *in vivo*

To investigate plasmid localization in living cells, first a plasmid containing an array of *lacO* operators (to be tagged by the chromosomally expressed LacI:GFP repressor) was constructed (BG1311 strain, see S1 Table). Unfortunately, the array of *lac* operators apparently affected plasmid replication in our genetic background, leading to gross rearrangements of a sub-population of cells. We therefore aimed to measure plasmid positioning by localizing the (ω:YFP)_2_-*parS* DNA complex.

The number of plasmid copies per cell was determined by quantitative PCR and also by hybridization upon normalizing to two distinct chromosomal genes. Under the growth conditions used there were on average, ~8 ± 1 plasmid copies/cell. This is in perfect agreement with previous data using the same replicon [36]. To analyze the dynamic localization of ω_2_ and/or δ_2_ during plasmid segregation *in vivo* we used three different systems that were grown asynchronously under slow growth rate conditions (in MMS7 medium) with a generation time of ~60 ± 5 min and at 30°C. In the first system the expression of plasmid borne ω and δ genes (or their variants) was controlled by ω_2_, (ω:YFP)_2_ or ω_2_ΔN19 (S3 Table).

**Fig 3:**
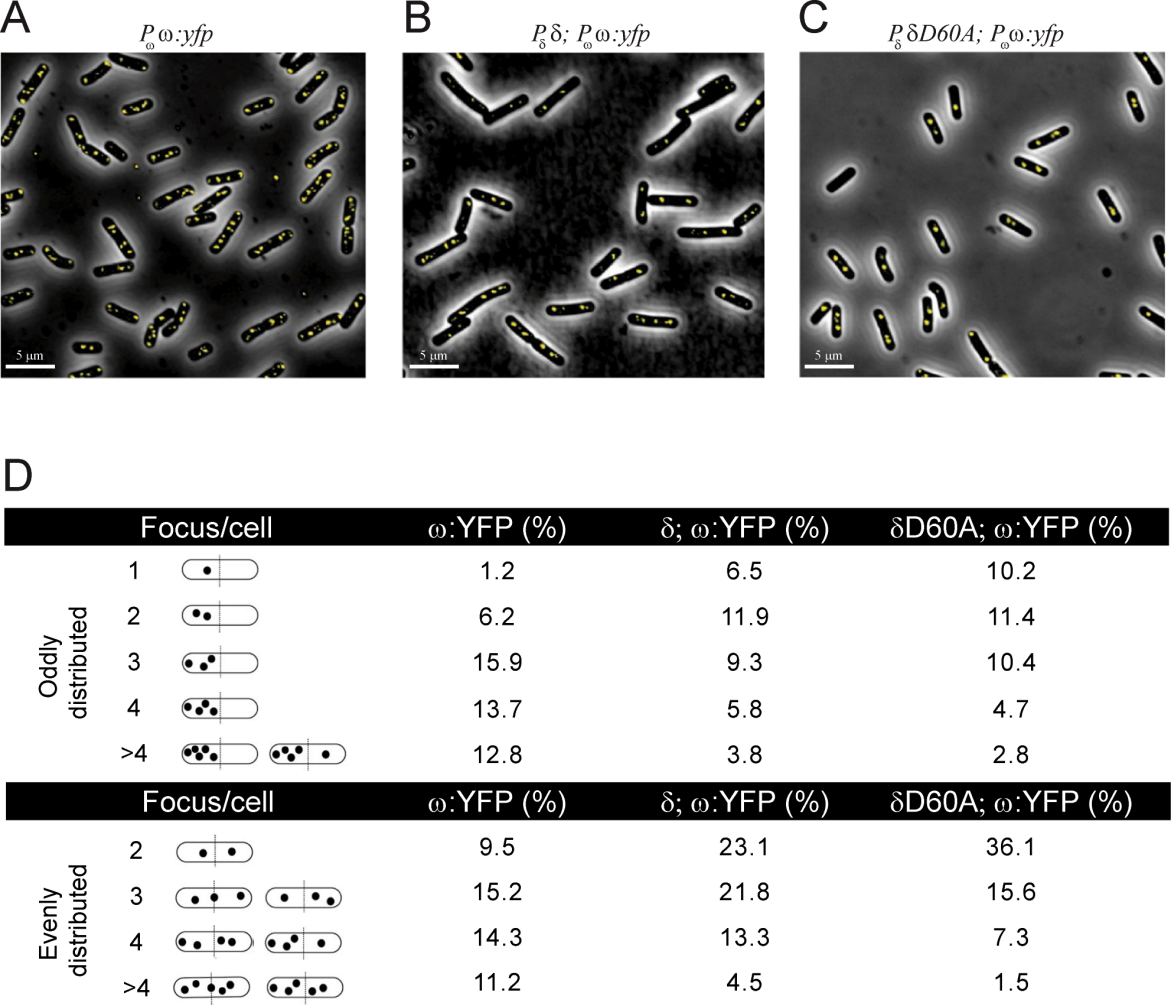
Subcellular position of (ω:YFP)_2_ foci in the absence or in the presence of δ_2_ or δ_2_D60A. Cells bearing plasmid-borne *P*_ω_ ω:*yfp* (A), *P*_δ_ δ and *P*_ω_ ω:*yfp* (B) or *P*_δ_ δD60A and *P*_ω_ ω:*yfp* genes (C) were grown in MMS7 at 30°C. YFP fluorescence of a typical field of each situation is presented. Scale bar is 5 μm. (D) The oddly or evenly distributed foci (1 to more than 4 foci) are shown schematically. The percentages of (ω:YFP)_2_ foci at each position around the cell center in the different conditions are indicated (calculated from >2,000 cells).

**Fig 4:**
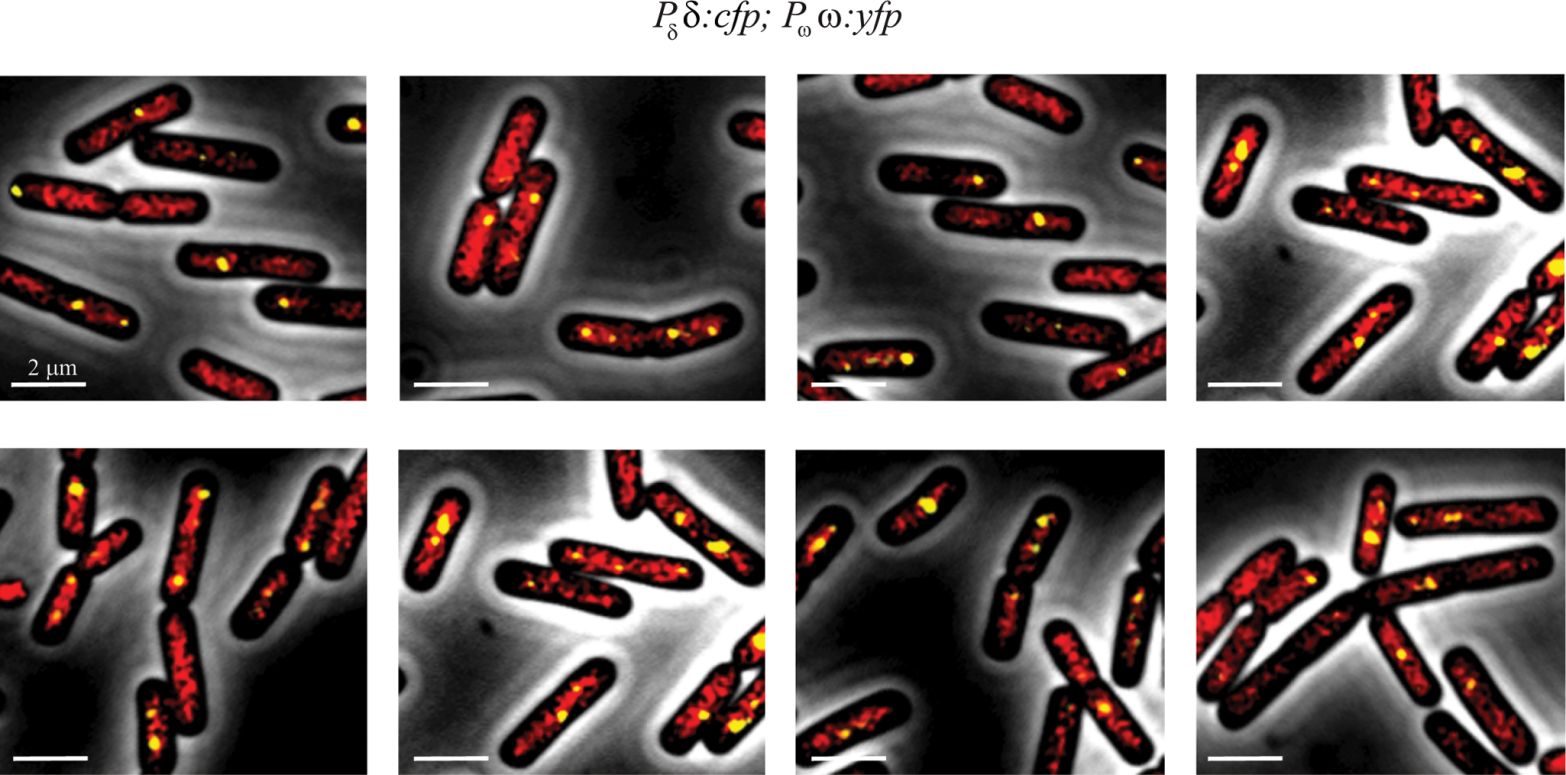
Subcellular co-localization of (δ:CFP)_2_ and (ω:YFP)_2_. Cells bearing plasmid-borne *P*_ω_ ω:*yfp* and *P*_δ_ δ:*cfp* genes were grown in MMS7 at 30°C. Images of the merged fluorescence from (δ:CFP)_2_ (in red) and (ω:YFP)_2_ (in yellow) are shown. Scale bar is 2 μm.

**Fig 5:**
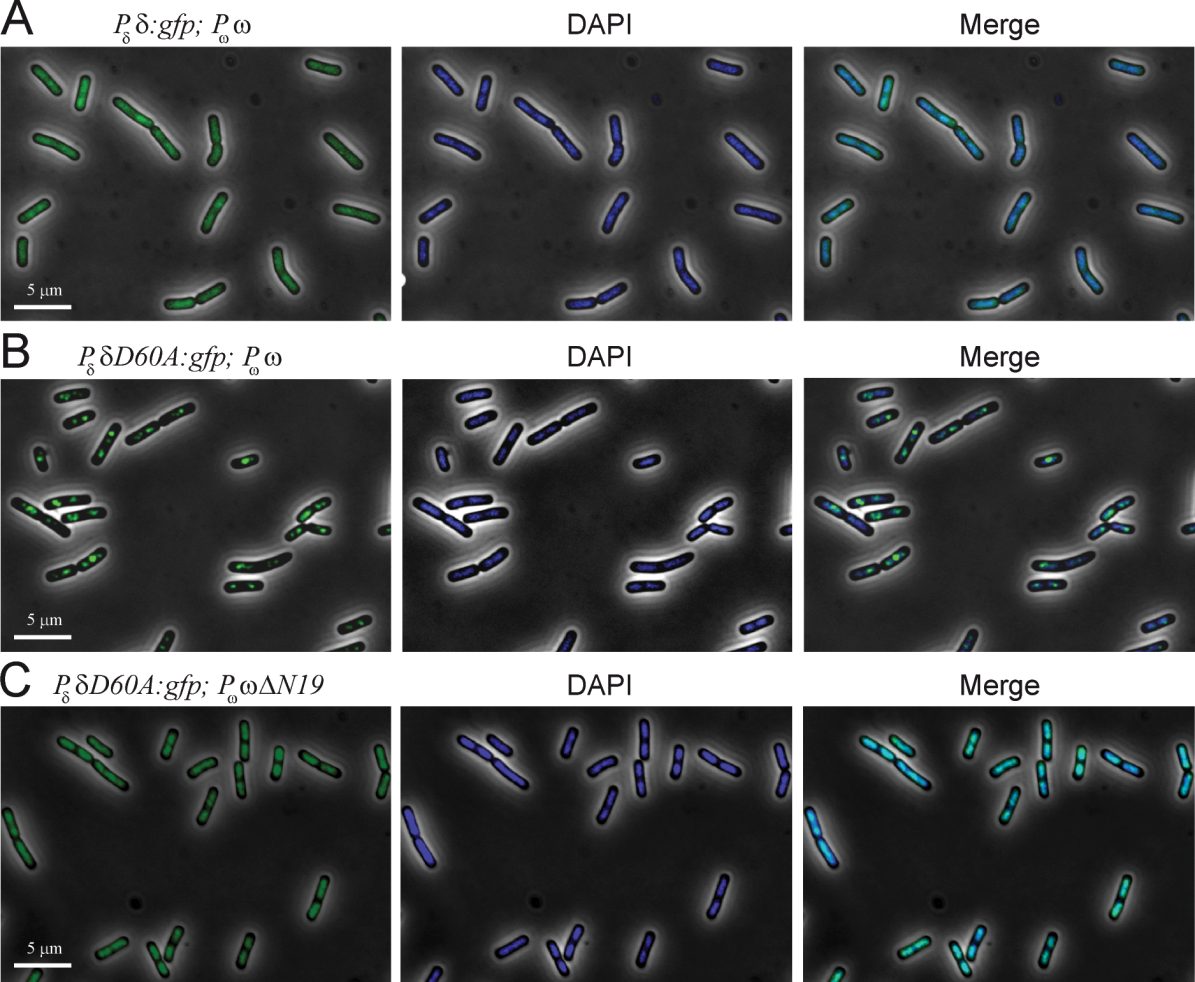
Subcellular position of (δ:GFP)_2_ or (δD60A:GFP)_2_ foci in presence of ω_2_ or ω_2_ΔN19. Cells bearing plasmid-borne *P*_δ_ δ:*gfp* and *P*_ω_ ω genes (A), *P*_δ_ δD60A:*gfp* and *P*_ω_ ω (B), or *P*_δ_ δD60A:*gfp* and *P*_ω_ ωΔN19 genes (C) were grown in MMS7 at 30°C. Images of cells with GFP fluorescence, images of the same cells stained with DAPI to show DNA, and the merge of both images are shown. Scale bar is 5 μm.

To determine the number of δ_2_ and ω_2_ molecules per cell, we performed quantitative immunoblots using anti-δ and anti-ω antibodies and purified δ_2_ and ω_2_ proteins as a standard. Our analysis, from at least four independent experiments, revealed that BG214 cells bearing a plasmid-borne *P*_δ_ δ (or *P*_δ_ δ:*gfp* or *P*_δ_ δD60A:*gfp*) and *P*_ω_ ω (or *P*_ω_ ω:*yfp* or *P*_ω_ ω_2_ΔN19), genes have ~1,400 ± 105 δ_2_ and ~1,300 ± 110 ω_2_ molecules/CFU (Table 1). Since the large majority of BG214 cells bearing plasmid were single- and two-cells clusters (with an average of 1.6 cells per CFU) (Figs 3–5), it was considered that each cell under controlled conditions contained 875 δ_2_ molecules (1.2 ± 0.1 μM) and 812 ω_2_ molecules (~ 1.1 ± 0.1 μM) and that the constructed variants had similar levels (Table 1). In the second system, plasmid-borne *P*_δ_ δ (or *P*_δ_ δ:*gfp*, *P*_δ_ δD60A or *P*_δ_ δD60A:*gfp*), in the absence of ω_2_ repression, was constitutively expressed from its native promoter, with ~7500 δ_2_ molecules/CFU or ~4600 molecules (6 μM)/cell (Table 1). In the third system, chromosomally encoded δ:*gfp* (or δD60A:*gfp*) was under the control of the LacI repressor, and its expression was induced by IPTG addition, whereas plasmid-borne ω_2_ (or ω_2_ΔN19) was controlled by its own promoter (*P*_ω_). Addition of 10 μM IPTG did not significantly change the average number of cells/CFU, and our analysis revealed that under these conditions there were ~ 6,500 (δ:GFP)_2_ or (δD60A:GFP)_2_ molecules/CFU or ~4000 δ_2_ and ~815 ω_2_ (or ω_2_ΔN19) molecules/cell (Table 1).

### Protein ω_2_ binds *parS* DNA and slightly facilitates plasmid clustering

The fluorescence of YFP-tagged ω_2_ expressed from its native promoter in asynchronous cells bearing plasmids harboring a *parS* site was analyzed (Fig 3A). In the absence of δ_2_, i.e. in plasmid-borne *P*_ω_ ω:*yfp*, (ω:YFP)_2_ formed bright foci. The (ω:YFP)_2_ fluorescence was neither homogeneously distributed on the cell cytosol nor formed clouds of fluorescence on the nucleoid (Fig 3).

The (ω:YFP)_2_ fluorescence formed discrete foci that were broadly distributed without showing any specific pattern (see Fig 3A and 3D). The distribution of (ω:YFP)_2_ fluorescence signal did not significantly vary with cell lengths. A similarly broad distribution was reported when the same replicon bearing an array of *lacO* sites and LacI-GFP was used [36]. It is likely, therefore, that the (ω:YFP)_2_ fluorescence highlights the location of the plasmid-borne *parS* site.

*In vi*tro, stoichometric ω_2_ concentrations have a footprint of ~70 ± 7 bp on *parS* DNA [16,30,31], and EM and AFM measurements revealed that there are 7 ± 1 ω_2_/*parS2* site, consisting of 7 contiguous iterons [8,27]. This is consistent with the observation that the volume of the (ω:YFP)_2_-*parS* foci is equivalent to one of the tau subunits of DNA polymerase (< 15 tau subunits/focus) [37]. The (ω:YFP)_2_ fluorescent foci never exceeded the number of plasmid copies (~8 ± 1/cell) (Fig 3A). As already mentioned, the total amount of (ω:YFP)_2_ in pCB846 bearing cells was ~800 ± 54 molecules/cell (Table 1). From these numbers and the number of (ω:YFP)_2_ fluorescent foci observed, we calculate that >85% of the (ω:YFP)_2_ molecules should be free in solution. Since the total fluorescence in the cells is provided by the (ω:YFP)_2_-*parS* foci, it was assumed that the free dimeric molecules do not assemble to give a quantitative fluorescence signal.

Further analysis of the fluorescence of (ω:YFP)_2_ revealed the presence of one to eight discrete (ω:YFP)_2_ foci/cell rather than patched structures (Fig 3A and 3D). More than 50% of the cells contained 4 or more foci per cell (Fig 3D). Since the number of plasmid origins (an indirect estimate of the number of plasmid copies) was not significantly altered during the experimental time and (ω:YFP)_2_ might not be a limiting factor, we assumed that (ω:YFP)_2_ might slightly facilitate plasmid clustering and that there were ~1.8 plasmid copies/focus. Alternatively, (ω:YFP)_2_ bound to *parS* may impair plasmid decatenation without altering the number of plasmid origins. We favor the former hypothesis, that individual protomers can contact sub-sites across *parS* sites. This is consistent with the observation that *in vitro* 8 to 10 ω_2_ molecules/plasmid facilitates plasmid bridging (pairing), albeit with low efficiency (~1% of total plasmid molecules) when analyzed by EM or by AFM [8,27]. Unlike the small ribbon-helix-helix (ω:YFP)_2_ protein (see Fig 3A and 3D), the helix-turn-helix large ParB-like proteins (represented by P1-ParB, F-SopB and *B*. *subtilis*-Sop0J), upon binding to *parS*, spread over nsDNA many kb to promote bridging, looping and condensation of nsDNA [10–12,15].

### Protein δ_2_ facilitates *in vivo* re-localization of the (ω:YFP)_2_-*parS* foci

To determine if the localization of the fluorescent foci was modified upon interaction of (ω:YFP)_2_ bound to *parS* with δ_2_, and to understand the role of ATP hydrolysis on this localization, the fate of the (ω:YFP)_2_-*parS* fluorescent foci was studied in the presence of wt δ_2_ or δ_2_D60A. In the presence of plasmid-borne *P*_δ_ δ and *P*_ω_ ω:*yfp* genes, there was a significant reduction in the number of fluorescent foci. The fluorescent foci re-localized toward cell quarters in bilobed cells, and at mid-cell in cells with one nucleoid (Fig 3B and 3D), suggesting that in the presence of δ_2_ the (ω:YFP)_2_-*parS* fluorescent foci might co-localize with the cell nucleoid. Similar results were observed when δ_2_ was replaced by the δ_2_D60A variant. This is consistent with the observation that: i) the (δ:GFP)_2_ (or δ_2_D60A) fluorescence co-localizes with the nucleoid (S1A and S1B Fig); and ii) (ω:YFP)_2_-*parS* fluorescent foci co-localized with the LacI-CFP bound to an the unstable array of plasmid-borne *lacO* sites (data not shown).

A quantification of >2,000 cells for each condition revealed that in the presence of wt δ_2_ or δ_2_D60A (which binds but does not hydrolyze ATP) there were <2 (ω:YFP)_2_ foci/cell in ∼41% or ∼58% of total cells (Fig 3B, 3C and 3D). However, in the absence of δ_2_ or δ_2_D60A only ∼17% of ∼2,000 total cells contained <2 (ω:YFP)_2_ foci/cell (Fig 3A and 3D). Since plasmid copy number was not significantly altered (∼8/cell) in any of the three conditions, we concluded that δ_2_ or δ2D60A promoted plasmid pairing (Fig 3D). On the other hand, in the presence of δ_2_D60A or δ_2_ only ∼16% and ∼27% of ∼2,000 total cells, respectively, contained 4 or more (ω:YFP)_2_ fluorescent foci/cell, whereas in the presence of only (ω:YFP)_2_ ∼52% of total cells contained 4 or more (ω:YFP)_2_ foci/cell (Fig 3D). These data are consistent with the *in vitro* observations that: i) δ_2_ and δ_2_D60A, upon interacting with ω_2_-*parS*, increased plasmid pairing with different frequency, with ∼20% of total complexes paired in the presence of wt δ_2_, and ∼60% of total complexes in the case of δ_2_D60A; and ii) in the absence of ATP hydrolysis (δ_2_D60A condition) the plasmids cannot unpair [8,27].

### Distribution of δ_2_ on the nucleoid

Previously we have shown that: i) δ_2_ binds nsDNA and forms discrete blobs (~5 ± 1 δ_2_/ blob) as seen by AFM, rather than bundles in the absence of any support, or filamentous structures on DNA [8]; ii) δ_2_D60A binds nsDNA with higher apparent affinity than wt δ_2_ (see S2 Table), because the δ_2_D60A-nsDNA complexes have a longer half-life than the wt δ_2_-nsDNA complexes [17]; and iii) (δK36A:GFP)_2_, which does not bind ATP, shows a fluorescence signal distributed in the cytosol [27]. This is consistent with absence of binding to nsDNA of apo-δ_2_ or ADP-bound δ_2_ *in vitro* (S2 Table).

When the fluorescence of (δ:GFP)_2_ was analyzed, it was found that it was regularly distributed over the nucleoid forming clouds of fluorescence, although low-density areas of fluorescence were observed (S1A and S2A Figs). Similar results were reported for P1-ParA and *Caulobacter crescentus* ParA *in vivo* [21,38].

When (δ:GFP)_2_ was replaced by (δD60A:GFP)_2_, it was found that, similar to the wt protein, the fluorescence was regularly distributed over the nucleoid, and low-density areas of fluorescence were also observed (S1B and S2B Figs). Since dynamic fluorescence on the nucleoid was observed with both proteins (S2 Fig), but (δD60A:GFP)_2_ cannot hydrolyze ATP, we favor the hypothesis that δ_2_ protein detachment from the nucleoid is independent of ATP hydrolysis.

*In vi*tro limiting δ_2_ concentrations have a footprint of ~30 ± 10 bp on nsDNA, and by AFM it was measured that a δ_2_ blob occupies ~80 ± 20 bp of nsDNA, and that there are ~5 ± 1 δ_2_/blob [8,27]. In the absence of ω_2_, there are ~4600 (δ-GFP)_2_ or (δD60A:GFP)_2_ molecules/cell, which drops to ~800 in the presence of ω_2_. Under this protein concentration, we are assuming the protein should be in its dimeric form, because a monomer does not bind nsDNA (data not shown). According to these data, we propose that (δ:GFP)_2_ or (δD60A:GFP)_2_ fluorescence is regularly distributed over the nucleoid, with less than 5% of the fluorescence located elsewhere (S1 and S2 Figs). Since >95% of the observed cloud of fluorescence is located on the nucleoid it is likely that there are ~800 (δ-GFP)_2_ or (δD60A:GFP)_2_ blobs/nucleoid(s) in the absence of ω_2_ and ~160 δ_2_ blobs/nucleoid(s) in the presence of ω_2_.

### Nucleoid bound (δ:CFP)_2_ captures and tethers (ω:YFP)_2_-*parS* copies

It has been shown that the plasmid replication machinery is highly mobile and predominantly located at or near the cell pole *in vivo* [36]. In the previous sections we have shown that: i) (δ:GFP)_2_ or (δD60A:GFP)_2_ fluorescence was apparently regularly distributed on the nucleoid (S1A and S1B Fig); ii) in the presence of δ_2_ or δD60A_2_ the (ω:YFP)_2_ fluorescent foci re-localized toward the middle of cells with one nucleoid, or toward cell quarters in bilobed cells; and iii) in the presence of δ_2_ or δD60A_2_ the number of (ω:YFP)_2_ foci was reduced, although the fluorescence signal per focus increased (Fig 3B and 3C). To study whether δ_2_ interaction with ω_2_ bound to *parS* DNA leads to capture and tethering of plasmid copies to the nucleoid, *P*_δ_ δ:*gfp* was replaced by *P*_δ_ δ:*cfp*, so that the localization of the two proteins could be studied simultaneously (i.e., plasmid-borne *P*_δ_ δ:*cfp* and *P*_ω_ ω:*yfp* genes were used, S1 Table). At or near physiological concentrations of both proteins, the cloud of (δ:CFP)_2_ fluorescence (denoted in red) formed on the nucleoid was not homogenously distributed, suggesting a certain dynamism (see below), and discrete (ω:YFP)_2_ fluorescent foci in the cytosol were not observed (Fig 4). (ω:YFP)_2_ formed 1 to 3 discrete foci at random positions on the nucleoid in ∼80% of the cells (Fig 4). The increased brightness of the (ω:YFP)_2_ fluorescent foci, as well as the reduced number of foci/cell observed suggested that many plasmids copies have been paired (Fig 4). Areas lacking the cloud of (δ:CFP)_2_ fluorescence also lacked the (ω:YFP)_2_ focus, suggesting that the ω_2_-*parS* complex on the nucleoid is a δ_2_-dependent reaction. To rationalize this observation, we propose that (δ:CFP)_2_, upon interaction with (ω:YFP)_2_, captures and tethers the plasmid molecules to (δ:CFP)_2_ on the nucleoid, leading to plasmid-nucleoid bridging (or pairing) (Fig 4). We propose that (ω:YFP)_2_, which fails to stimulate (δ:CFP)_2_ ATPase activity, will lead to accumulation of these bridging intermediates.

### *parS*-bound ω_2_ stimulates δ_2_ disassembly from the nucleoid

**Fig 6:**
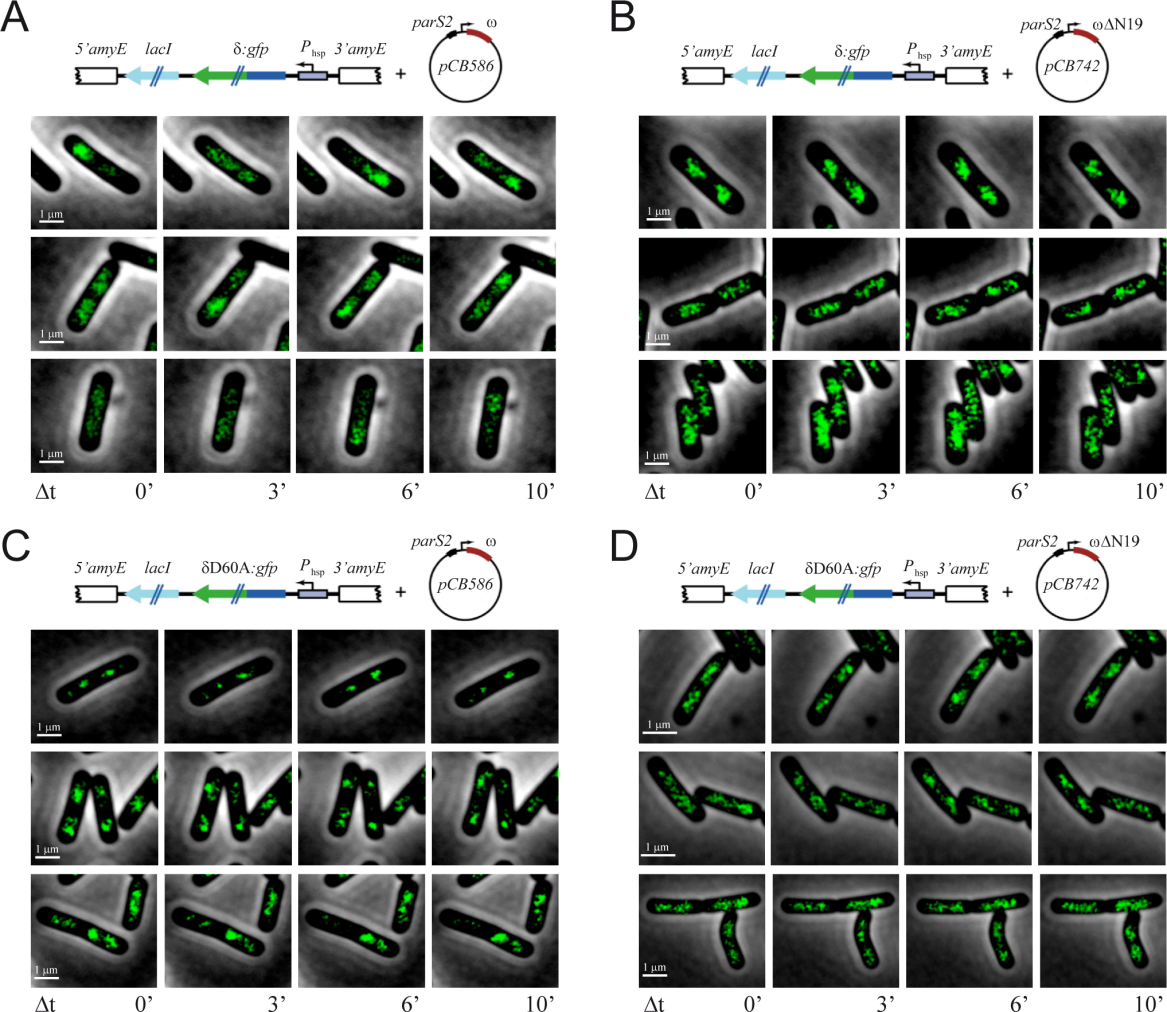
Time lapse of (δ:GFP)_2_ or (δD60A:GFP)_2_ at unbalanced levels in the presence of ω_2_ or ω_2_ΔN19. Cells had the *P*_lac_ δ:*gfp* (A and B) or *P*_lac_ δD60A:*gfp* (C and D) genes integrated into the *B*. *subtilis* chromosome, and plasmid-borne *P*_ω_ ω (pCB586) (A and C) or *P*_ω_ ωΔN19 gene (pCB742) (B and D). Images of the same cells with GFP fluorescence from (δ:GFP)_2_ or (δD60A:GFP)_2_ are shown for the indicated times. Scale bar is 1 μm.

The dynamic change that the ω_2_:δ_2_ interaction may promote in (δ:GFP)_2_ or (δD60A:GFP)_2_ localization was analyzed at or near physiological concentrations of both proteins. In the presence of both ω_2_ and (δ:GFP)_2_, the (δ:GFP)_2_ fluorescence was more irregularly distributed on the nucleoid when compared to the absence of ω_2_ (Fig 5A *vs* S1A Fig), suggesting that after (δ:GFP)_2_ detachment from the nucleoid, fluorescence in the cytosol was not observed (Fig 5A). Alternatively, the areas of low fluorescence observed here were simply due to the fact that ω_2_ repressed δ:*gfp* expression and there was not sufficient protein to produce the cloudiness on the nucleoid. To test this hypothesis, the (δ:GFP)_2_ concentration was artificially increased in the background, but the same outcome was observed (Fig 6A).

In the presence of ω_2_, (δD60A:GFP)_2_, which binds but cannot hydrolyze ATP, lost its regular distribution over the nucleoid, and it accumulated as discrete foci or patched regions on the nucleoid (Fig 5B). A comparison of Fig 5B and S1B Fig revealed that the (δD60A:GFP)_2_ fluorescence detached from the nucleoid even in the absence of ATP hydrolysis, and that the foci or patched regions may correspond to non-disassembled plasmid-nucleoid bridging complexes. This is consistent with the observation that δ_2_D60A and ω_2_ lead to accumulation of bridging intermediates *in vitro* [8].

To confirm that the areas of low fluorescence observed here are not simply due to the lower (δD60A:GFP)_2_, concentration we performed experiments in the presence of ω_2_ΔN19, which also represses δD60A:*gfp* expression (S3 Table), but fails to interact with δ_2_ [32]. In the presence of ω_2_ΔN19, the fluorescence of (δD60A:GFP)_2_ dramatically changed, and was indistinguishable from that observed in the presence of (δD60A:GFP)_2_ alone (i.e, in the absence of ω_2_ΔN19, Fig 5C *vs* S1B Fig). Similar results were observed when (δD60A:GFP)_2_ was replaced by δ_2_ in the presence of ω_2_ΔN19 (data not shown). It is likely that the interaction of ω_2_ with (δ:GFP)_2_ or (δD60A:GFP)_2_ dramatically changed their pattern of fluorescence.

### Dynamic redistribution of δ_2_ on the nucleoid

To further determine the type of complexes that (δ:GFP)_2_ or (δD60A:GFP)_2_ could form, the δ_2_ concentration was artificially raised and uncoupled from ω_2_ expression. The δ:*gfp* or δD60A:*gfp* gene was transcribed from the IPTG-inducible promoter (*P*_hsp_), and integrated as a unique copy at the host *amyE* locus (S1 Table). In the presence of 10 μM IPTG, there were ~4,000 molecules (δ:GFP)_2_ or (δD60A:GFP)_2_/cell (Table 1). Under this experimental condition plasmid segregation was not significantly affected, but in the presence of 50 μM IPTG plasmid partitioning is significantly impaired (S4 Table) [28]. Hence, the former condition was used for further analyses.

In the absence of ω_2_, the fluorescence of (δ:GFP)_2_ or (δD60A:GFP)_2_ was regularly distributed over the nucleoid (S3A and S3B Fig), and these clouds of fluorescence by (δ:GFP)_2_ or (δD60A:GFP)_2_ showed dynamic behavior in a time-dependent manner (S3A and S3B Fig), suggesting that protein disassembly from the nucleoid does not require hydrolysis of ATP. The presence of physiological ω_2_ concentrations significantly increased the dynamism of the cloud of fluorescence (Fig 6A and 6C). Time-lapse microscopy, with images gathered every 20 s over a 10 min period, were carried out in cells growing as micro-colonies on a slide. A re-organization and decrease in the level of (δ:GFP)_2_ fluorescence at a given location in the presence of physiological ω_2_ was taken as an indirect measure of (δ:GFP)_2_ disassembly from the nucleoid, rather than no assembly. This is consistent with the observation that in the absence of ω_2_ or in the presence of ω_2_ΔN19 the fluorescence was more regularly distributed over the nucleoid (Fig 6A *vs* Fig 6B or S3A Fig). It is likely that the interaction of (δ:GFP)_2_ bound to the nucleoid with a ω_2_-*parS* complex stimulates the (δ:GFP)_2_ ATPase, and (δ:GFP)_2_-ADP might lose affinity for DNA. This is consistent with the observation that δ_2_-ADP showed a very low affinity for nsDNA (S2 Table).

In the presence of ω_2_, (δD60A:GFP)_2_ formed discrete foci or patched regions on the nucleoid (Fig 6C). To explain this pattern of (δD60:GFP)_2_ fluorescence and its partitioning disability, we propose that δ_2_D60A failed to promote ATP hydrolysis-dependent plasmid-nucleoid disassembly, but still (δD60:GFP)_2_ redistributed on the nucleoid in the absence of ATP hydrolysis (Fig 6C). It is likely that ω_2_-mediated stimulation of (δD60:GFP)_2_ relocation is unlinked from plasmid movement, because discrete foci or patched regions were attributed to the accumulation of tethered plasmids. However, when ω_2_ was replaced by ω_2_ΔN19, the amount of (δD60:GFP)_2_ was not modified but the fluorescence was regularly distributed over the nucleoid (Fig 6D). This is consistent with the observation that ATP hydrolysis is necessary for disassembly of quaternary complexes (*parS*-ω_2_-δ_2_-nsDNA) or plasmid unpairing [8], but it is not essential to redistribute δ_2_ on the nucleoid. Similar results were reported for F-SopA (see [39]).

### Protein δ_2_ variants impaired in nsDNA binding show a complex phenotype

**Fig 7:**
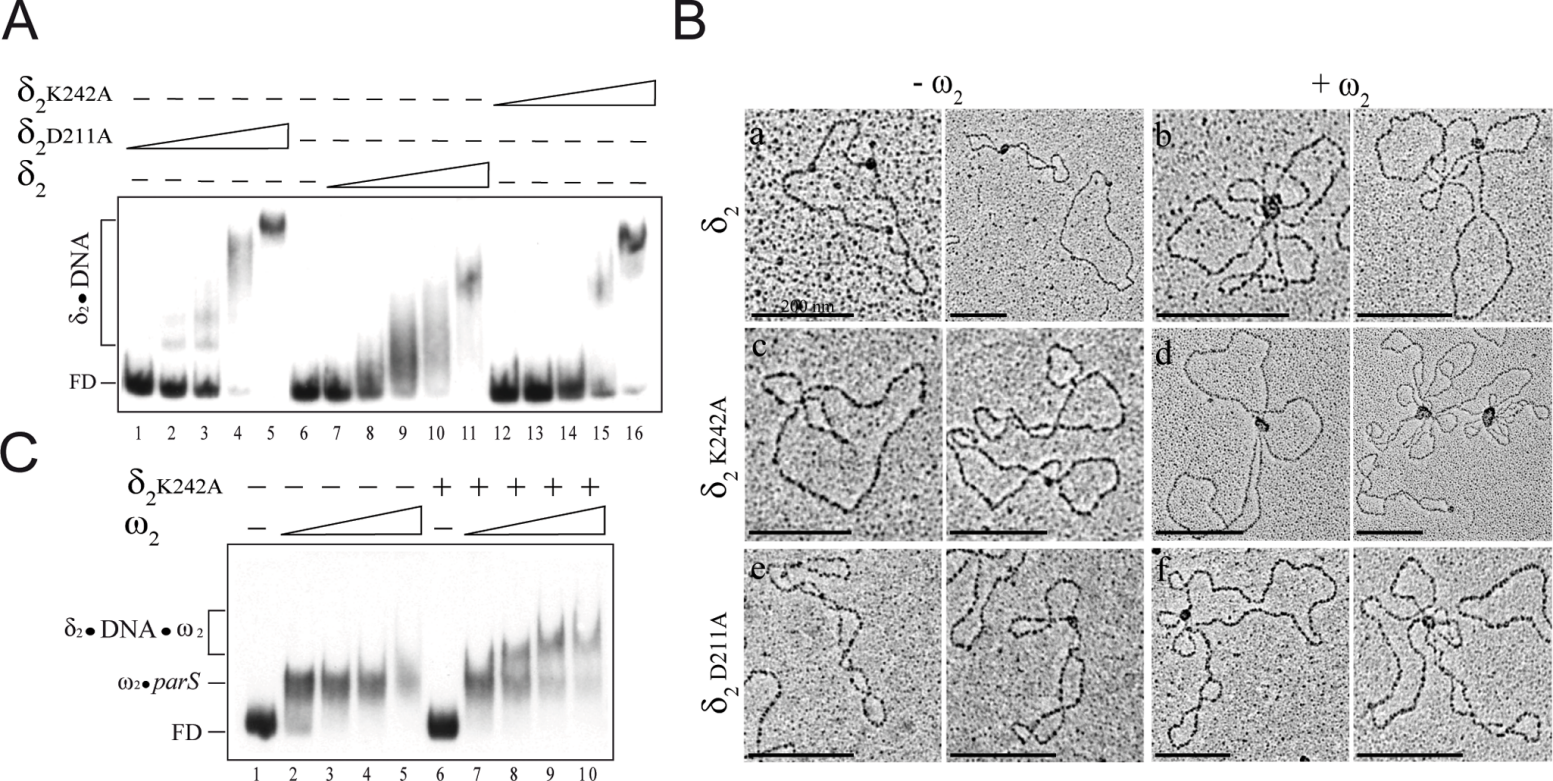
Protein δ_2_K242A binds poorly to DNA forming transient complexes. (A) DNA binding affinity of the different δ_2_ variants. The 423-bp [α^32^P]-*parS* DNA (0.1 nM) was incubated with increasing concentrations of δ_2_ (37, 75, 150, 300 and 600 nM), δ_2_D211A (4, 9, 18, 17 and 75 nM) or δ_2_K242A (300, 600, 1200, 2400 and 4800 nM) in buffer C containing 1 mM ATP. The free DNA (FD) and the formed complexes are indicated. (B) Electron micrographs of protein-DNA complexes and plasmid pairing observed in the presence of δ_2_ variants and ω_2_. pCB30 DNA (*parS*2) was incubated in the presence of 1 mM ATP with δ_2_ (150 nM), δ_2_K242A (300 nM) or δ_2_D211A (75 nM) and ω_2_ (60 nM) when indicated and the complexes formed were visualized by EM. Scale bars in black indicate 200 nm. (C) Protein ω_2_ facilitates the loading of δ_2_K242A onto *parS* DNA. The 423-bp [α^32^P]-*parS* DNA (0.1 nM) was pre-incubated with increasing concentrations of ω_2_ (6 to 48 nM) or with a fixed concentration of δ_2_K242A (300 nM) and then increasing concentrations of ω_2_. Reactions were performed in buffer C containing 1 mM ATP.

Exchange of a single positively charged residue in δ_2_ (e.g., K242, Fig 1D) to alanine resulted in a 20-fold decrease in binding efficiency to nsDNA *in vitro* (Fig 7A, S2 Table) [17], but this mutation only reduced plasmid stability by to 2- to 3-fold (Fig 2). In contrast, exchange of a single negatively charged residue in δ_2_ (e.g., D211) to alanine resulted in a 6-fold increase in binding efficiency to nsDNA *in vitro* (Fig 7A, S2 Table) without affecting faithful plasmid segregation (Fig 2). These data are in apparent contradiction with the prevailing partitioning model, and negate the requirement for ParA binding to nsDNA for efficient partitioning [40–41]. To explain these results, the complexes formed by these δ_2_ variants upon binding to nsDNA were analyzed by EMSA and EM. Protein δ_2_K242A or δ_2_D211A binds and catalyzes the hydrolysis of ATP to an extent similar to wt δ_2_ (A.V., unpublished results). Protein δ_2_, in the ATP-bound form, cooperatively binds nsDNA with K_Dapp_ ~140 nM, but δ_2_K242A seemed to fail to form stable complexes with nsDNA even in the presence of 1200 nM (Fig 7A and S2 Table). However, δ_2_K242A formed protein-DNA complexes in the presence of a ∼20-fold excess relative to the wt δ_2_ K_Dapp_ (Fig 7A, lanes 15–16 and S2 Table) [17]. Therefore, we analyzed whether the δ_2_K242A mutation was still able to form a complex with nsDNA *in vivo*. In the absence of ω_2_, (δK242A:GFP)_2_ formed a regular cloud of fluorescence over the nucleoid, indistinguishable from the one observed with (δ:GFP)_2_. However, when the cells were not fixed with paraformaldehyde before visualization, a large fraction of cells contained the fluorescence distributed into the cytosol (data not shown). It is likely that (δK242A:GFP)_2_ forms transient complexes on nsDNA, but a cooperative interaction with ω_2_ might ameliorate this defect *in vivo* because plasmid segregation was only marginally affected (Fig 2). To test this hypothesis, EM experiments were performed. In the presence of limiting protein concentrations (∼10-fold below K_Dapp_, 300 nM, S2 Table), δ_2_K242A assembled to form one discrete blob per DNA molecule in ~37% of the DNA molecules (*n* = 530) (Fig 7Bc). When the DNA was linearized the δ_2_K242A blobs showed a random location, which indicated non-specific binding to DNA (data not shown). Similar types of complexes were observed in the presence of wt δ_2_ or the δ_2_D211A variant (Fig 7Ba and 7Be). As previously documented [8,17], intermolecular bridging of two plasmid molecules by δ_2_, δ_2_K242A, or δ_2_D211A was not observed (*n* = 530, 440 and 460, respectively) (Fig 7B).

Previously it was shown by AFM that ω_2_-*parS* complexes (220 ± 56 nm^3^) are smaller in size than the δ_2_-nsDNA (430 ± 42 nm^3^) complexes, and both complexes are smaller than the quaternary complex (*parS*-ω_2_-δ_2_-*parS*, 800 ± 100 nm^3^) [8,27]. In the presence of ω_2_, δ_2_-mediated clusters larger than the protein alone were observed in ∼80% of the *parS* DNA molecules and plasmid pairing in the remaining ~20% (*n* = 456) (Fig 7Bb). Similar results were observed when δ_2_ was replaced by δ_2_D211A (Fig 7Bf). In the presence of preformed ω_2_-*par*S complexes δ_2_K242A only co-localized with ω_2_ bound to *par*S and facilitated plasmid pairing formation in ∼42% (*n* = 86) of the DNA molecules (Fig 7Bd). When the ω_2_:δ_2_K242A ratio was reduced, protein clusters were observed in ∼62% of the DNA molecules, and ∼ 18% of the *parS* DNA molecules were paired with DNA molecules juxtaposed at their ω_2_-*parS* DNA (*n* = 586). Together these data suggest that δ_2_K242A at 300 nM formed transient complexes that cannot be detected by EMSA (Fig 7A), but can be visualized by EM (Fig 7B). To confirm that the presence of ω_2_ increases the stability of δ_2_K242A-nsDNA complexes by decreasing the off rate of δ_2_K242A binding to DNA, EMSA experiments were performed in the presence of various ω_2_ concentrations. The presence of ω_2_ and limiting δ_2_K242A (300 nM) facilitated the formation of a slow-mobility ternary ω_2_-*parS* DNA-δ_2_K242A complex (Fig 7C), but this ternary complex was not observed if ATP was omitted (S2 Table, data not shown). It is likely that: i) the ω_2_-*parS* DNA complexes stabilized the δ_2_K242A-DNA complex to form ternary (*parS*-ω_2_-δ_2_K242A) and quaternary (*parS*-ω_2_-δ_2_K242A-ω_2_-*parS*) complexes; and ii) δ_2_ binding to the nucleoid is a crucial step in accurate plasmid partitioning.

## Discussion

Plasmid pSM19035 partitioning, which uses to the non-filament-based mode, depends on the dynamic interaction among the δ_2_ ATPase bound to chromosomal DNA, the ω_2_ CBP and the *parS* sites. It is a multi-step process with discrete functional transitions. First, plasmid replication occurs mostly at nucleoid-free regions (e.g., at the cell poles) and occasionally moves out of them in the absence of ParAB [36]. In the presence of only the small ParB-like ω_2_ protein, binding to the *parS* region in the newly replicated plasmid leads to moderate plasmid clustering (Fig 3A). This finding is consistent with previous data showing that two ω_2_-*parS* complexes form transient cluster intermediates, and ω_2_-mediated clustering accounts only for ∼1% of total protein-DNA complexes *in vitro* by EM or AFM analyses [8,17,27]. In contrast, the large helix-turn-helix ParB proteins (e.g., P1-ParB, F-SopB or chromosomal-encoded Spo0J), upon binding to *parS*, spread over nsDNA many kb and promote bridging (pairing), looping and condensation of nsDNA [10–12,15].

Second, in the presence of only δ_2_, this small ATPase binds dynamically to nsDNA (i.e, the nucleoid) in a process where ATP binding, but not hydrolysis, is essential; constitutive (δ:GFP)_2_ or (δD60A:GFP)_2_ expression led to a dynamic cloud of fluorescence on the nucleoid (S3 Fig). Unlike pB171-ParA [42], we did not detect oscillation of (δ:GFP)_2_ or (δD60A:GFP)_2_ from pole to pole. It was estimated that under constitutive expression there are ∼800 (δ:GFP)_2_ or (δD60A:GFP)_2_ blobs/cells (see Table 1). Since a fluorescence signal of free (δ:GFP)_2_ in solution was not detected (see S1–S3 Figs), it is likely that bundle structures were not formed in solution (S3 Fig). A dynamic cloud of fluorescence with slow detachment was observed in constitutively or LacI-regulated (δ:GFP)_2_ or (δD60A:GFP)_2_, e.g., in the presence or the absence of ATP hydrolysis, respectively (S3 Fig). Similarly, F-SopA relocation is not impeded by severely weakened ATP hydrolysis [39]. In the large ParA ATPases (e.g., P1-ParA or F-SopA) the reaction is more complex. Here, ParA binds and hydrolyses ATP and this enables ParA-ADP to bind specific DNA sequences required to regulate the expression of the ParAB locus; in addition, binding to ATP without hydrolysis produces a slow conformational transition in ParA that enables it to bind nsDNA and form a carpet on the DNA [4,6].

Third, the interaction of *parS*-ω_2_ complexes with δ_2_ at the nucleoid relocates the plasmid copies from a broad distribution towards the high concentration of δ_2_ bound to the nucleoid (plasmid-nucleoid pairing) (Fig 4). This plasmid capturing and tethering is consistent with the fact that the interaction of ω_2_ with δ_2_ enhances binding of the latter to nsDNA, and facilitates plasmid-nucleoid pairing (Fig 7). When ω_2_ was replaced by ω_2_ΔN19, the plasmids freely diffuse, leading to random segregation (Fig 2), suggesting that δ_2_ bound to the nucleoid captures, moves and tethers plasmid-borne ω_2_-*parS* by interaction with ω_2_.

Fourth, the dynamic δ_2_-ω_2_ interactions at the paired complexes should alter the relative stoichiometry of both proteins. *In vitro*, the δ_2_ ATPase activity was maximal at ~1.5:1 ω_2_:δ_2_ ratios [27], and this may correspond to fluorescence depleted zones (Figs 5 and 6). This is consistent with the *in vitro* observation that δ_2_·ADP promotes plasmid unpairing and it enhances dissociation of δ_2_ from the nsDNA (the nucleoid) [27]. However, to explain the slow δ_2_ or (δD60A:GFP)_2_ re-association with the nucleoid, we have to assume that ω_2_ may induce a conformational transition in δ_2_ or in (δD60A:GFP)_2_ that weakens its re-assembly onto the nucleoid, as seen *in vitro* [23].

Fifth, the transient disassembly of δ_2_ from the plasmid-nucleoid complex should increase the relative concentration of ω_2_. This hypothesis is based on the observation that when both proteins are present at about stoichiometric concentrations, disassembly of δ_2_ increases, because its ATPase activity is activated. The individual ω_2_-*parS* complexes (i.e, the individual plasmids) should then ratchet along the newly formed cloud of δ_2_-nsDNA that could be seen as a cargo moving daughter plasmids away from each other over the surface of the nucleoid and re-pairing in a distant location on the nucleoid, following an oscillating wave of δ_2_ binding and release from the nucleoid. By this dynamic process, the ω_2_-*parS* complexes could actively move towards the newly separated nucleoids, so that at cell division, each daughter cell should receive at least one plasmid copy. Finally, the accumulation of discrete foci and patched structures observed with ω_2_-δ_2_D60A suggests that ATP hydrolysis is required for plasmid unpairing, and this defect contributes to the impairment in plasmid partitioning (Fig 2). This is consistent with the *in vivo* data that showed that (δD60A:GFP)_2_ redistributed on the nucleoid in the presence of ω_2_ (Figs 5B and 6C), resulting in increased plasmid pairing (Fig 3), and with *in vitro* data showing that *parS*-ω_2_-δ_2_D60A-ω_2_-*parS* complexes cannot disassemble, but the *parS*-ω_2_-δ_2_-ω_2_-*parS* complexes can easily become unpaired [8]. This may effectively bias plasmid random diffusion toward the cell quarters, resulting in accurate plasmid segregation. The data presented in this work are supporting the non-filament-based modes of partitioning, which is the mode proposed for the large P1-ParAB-*parS* or F-SopAB-*sopC* [6,22,24] or the large/small *C*. *crescentus* ParBA-*parS* system [25].

## Supporting Information

S1 Fig Subcellular localization of (δ:GFP)_2_ or (δD60A:GFP)_2_. Cells bearing plasmid-borne *P*_δ_ δ:*gfp* (A) or *P*_δ_ δD60A·*gfp* gene (B) were grown in MMS7 at 30°C. Fluorescence images of cells, images of the same cells stained with DAPI to show DNA, and the merge of both images are shown. Scale bar is 5 μm.
(PDF)

S2 Fig Time lapse of (δ:GFP)_2_ or (δD60A:GFP)_2_ fluorescence. Cells bearing-plasmid borne *P*_δ_ δ:*gfp* (A) or *P*_δ_ δD60A:*gfp* gene (B) were grown in MMS7 at 30°C. Images of the same cells with fluorescence from (δ·GFP)_2_ or (δD60A·GFP)_2_ are shown for the indicated time. Scale bar is 5 μm.
(PDF)

S3 Fig Subcellular localization of (δ:GFP)_2_ or (δD60A:GFP)_2_. Illustration showing the structure of the *P*_*hsp*_ *δ*:*gfp* (A) or *P*_*hsp*_ *δD60A*:*gfp* (B) expression cassettes integrated in the host chromosome, rendering strains BG947 and BG1097, respectively. Cells were grown in MMS7 at 30°C in the presence of 10 μM IPTG. GFP fluorescence images of cells, images of the same cells stained with DAPI to show DNA, and the merge of both images are shown. Scale bar is 5 μm.
(PDF)

S1 Table Strains and plasmids. (DOCX)

S2 Table Relative binding of δ_2_ or its variants to nsDNA. (DOCX)

S3 Table Protein (ω:YFP)_2_ binds *P*_δ_ and represses transcription. (DOCX)

S4 Table Effect of ω:*yfp* expression in faithful segregation or plasmid incompatibility. (DOCX)

## References

[1] Reyes-Lamothe R, Nicolas E, Sherratt DJ (2012) Chromosome replication and segregation in bacteria. Annu Rev Genet 46: 121–143. 10.1146/annurev-genet-110711-155421 22934648

[2] Wang X, Montero Llopis P, Rudner DZ (2013) Organization and segregation of bacterial chromosomes. Nat Rev Genet 14: 191–203. 10.1038/nrg3375 23400100 PMC3869393

[3] Gerdes K, Moller-Jensen J, Bugge Jensen R (2000) Plasmid and chromosome partitioning: surprises from phylogeny. Mol Microbiol 37: 455–466. 10931339 10.1046/j.1365-2958.2000.01975.x

[4] Davey MJ, Funnell BE (1994) The P1 plasmid partition protein ParA. A role for ATP in site-specific DNA binding. J Biol Chem 269: 29908–29913. 7961987

[5] Dunham TD, Xu W, Funnell BE, Schumacher MA (2009) Structural basis for ADP-mediated transcriptional regulation by P1 and P7 ParA. EMBO J 28: 1792–1802. 10.1038/emboj.2009.120 19461582 PMC2699355

[6] Vecchiarelli AG, Han YW, Tan X, Mizuuchi M, Ghirlando R, Biertumpfel C, et al (2010) ATP control of dynamic P1 ParA-DNA interactions: a key role for the nucleoid in plasmid partition. Mol Microbiol 78: 78–91. 10.1111/j.1365-2958.2010.07314.x 20659294 PMC2950902

[7] Ringgaard S, van Zon J, Howard M, Gerdes K (2009) Movement and equipositioning of plasmids by ParA filament disassembly. Proc Natl Acad Sci U S A 106: 19369–19374. 10.1073/pnas.0908347106 19906997 PMC2775997

[8] Pratto F, Suzuki Y, Takeyasu K, Alonso JC (2009) Single-molecule analysis of proteinxDNA complexes formed during partition of newly replicated plasmid molecules in Streptococcus pyogenes. J Biol Chem 284: 30298–30306. 10.1074/jbc.M109.035410 19726689 PMC2781585

[9] Schumacher MA, Ye Q, Barge MT, Zampini M, Barilla D, Hayes F (2012) Structural Mechanism of ATP-induced Polymerization of the Partition Factor ParF: Implication for DNA segregation. J Biol Chem 287: 26146–26154. 10.1074/jbc.M112.373696 22674577 PMC3406698

[10] Lynch AS, Wang JC (1995) SopB protein-mediated silencing of genes linked to the *sopC* locus of *Escherichia coli* F plasmid. Proc Natl Acad Sci U S A 92: 1896–1900. 7534407 10.1073/pnas.92.6.1896 PMC42389

[11] Rodionov O, Lobocka M, Yarmolinsky M (1999) Silencing of genes flanking the P1 plasmid centromere. Science 283: 546–549. 9915704 10.1126/science.283.5401.546

[12] Murray H, Ferreira H, Errington J (2006) The bacterial chromosome segregation protein Spo0J spreads along DNA from parS nucleation sites. Mol Microbiol 61: 1352–1361. 16925562 10.1111/j.1365-2958.2006.05316.x

[13] Havey JC, Vecchiarelli AG, Funnell BE (2012) ATP-regulated interactions between P1 ParA, ParB and non-specific DNA that are stabilized by the plasmid partition site, *parS* . Nucl Acids Res 40: 801–812. 10.1093/nar/gkr747 21965538 PMC3258138

[14] Schumacher MA, Funnell BE (2005) Structures of ParB bound to DNA reveal mechanism of partition complex formation. Nature 438: 516–519. 16306995 10.1038/nature04149

[15] Graham TG, Wang X, Song D, Etson CM, van Oijen AM, Rudner DZ et al (2014) ParB spreading requires DNA bridging. Genes Dev 28: 1228–1238. 10.1101/gad.242206.114 24829297 PMC4052768

[16] Weihofen WA, Cicek A, Pratto F, Alonso JC, Saenger W (2006) Structures of ω repressors bound to direct and inverted DNA repeats explain modulation of transcription. Nucl Acids Res 34: 1450–1458. 16528102 10.1093/nar/gkl015 PMC1401508

[17] Soberón NE, Lioy VS, Pratto F, Volante A, Alonso JC (2011) Molecular anatomy of the *Streptococcus pyogenes* pSM19035 partition and segrosome complexes. Nucl Acids Res 39: 2624–2637. 10.1093/nar/gkq1245 21138966 PMC3074150

[18] Barilla D, Rosenberg MF, Nobbmann U, Hayes F (2005) Bacterial DNA segregation dynamics mediated by the polymerizing protein ParF. EMBO J 24: 1453–1464. 15775965 10.1038/sj.emboj.7600619 PMC1142544

[19] Ptacin JL, Lee SF, Garner EC, Toro E, Eckart M, Comolli LR et al (2010) A spindle-like apparatus guides bacterial chromosome segregation. Nature Cell Biol 12: 791–798. 10.1038/ncb2083 20657594 PMC3205914

[20] Hatano T, Niki H (2010) Partitioning of P1 plasmids by gradual distribution of the ATPase ParA. Mol Microbiol 78: 1182–1198. 10.1111/j.1365-2958.2010.07398.x 21091504

[21] Sengupta M, Nielsen HJ, Youngren B, Austin S (2010) P1 plasmid segregation: accurate redistribution by dynamic plasmid pairing and separation. J Bacteriol 192: 1175–1183. 10.1128/JB.01245-09 19897644 PMC2820840

[22] Hwang LC, Vecchiarelli AG, Han YW, Mizuuchi M, Harada Y, Funnell BE et al (2013) ParA-mediated plasmid partition driven by protein pattern self-organization. EMBO J 32: 1238–1249. 10.1038/emboj.2013.34 23443047 PMC3642677

[23] Vecchiarelli AG, Hwang LC, Mizuuchi K (2013) Cell-free study of F plasmid partition provides evidence for cargo transport by a diffusion-ratchet mechanism. Proc Natl Acad Sci U S A 110: E1390–1397. 10.1073/pnas.1302745110 23479605 PMC3625265

[24] Vecchiarelli AG, Neuman KC, Mizuuchi K (2014) A propagating ATPase gradient drives transport of surface-confined cellular cargo. Proc Natl Acad Sci U S A 111: 4880–4885. 10.1073/pnas.1401025111 24567408 PMC3977271

[25] Lim HC, Surovtsev IV, Beltran BG, Huang F, Bewersdorf J, Jacobs-Wagner C (2014) Evidence for a DNA-relay mechanism in ParABS-mediated chromosome segregation. Elife 3: e02758 10.7554/eLife.02758 24859756 PMC4067530

[26] Lioy VS, Pratto F, de la Hoz AB, Ayora S, Alonso JC (2010) Plasmid pSM19035, a model to study stable maintenance in Firmicutes. Plasmid 64: 1–17. 10.1016/j.plasmid.2010.04.002 20403380

[27] Pratto F, Cicek A, Weihofen WA, Lurz R, Saenger W, Alonso JC (2008) *Streptococcus pyogenes* pSM19035 requires dynamic assembly of ATP-bound ParA and ParB on *parS* DNA during plasmid segregation. Nucl Acids Res 36: 3676–3689. 10.1093/nar/gkn170 18477635 PMC2441792

[28] Pratto F (2007) Análisis del sistema de partición activa del plásmido pSM19035 de Streptococcus pyogenes. Madrid: Universidad Autónoma de Madrid 100 p.

[29] Murayama K, Orth P, de la Hoz AB, Alonso JC, Saenger W (2001) Crystal structure of ω transcriptional repressor encoded by *Streptococcus pyogenes* plasmid pSM19035 at 1.5 Å resolution. J Mol Biol 314: 789–796. 11733997 10.1006/jmbi.2001.5157

[30] de la Hoz AB, Ayora S, Sitkiewicz I, Fernandez S, Pankiewicz R, Alonso JC (2000) Plasmid copy-number control and better-than-random segregation genes of pSM19035 share a common regulator. Proc Natl Acad Sci U S A 97: 728–733. 10639147 10.1073/pnas.97.2.728 PMC15398

[31] de la Hoz AB, Pratto F, Misselwitz R, Speck C, Weihofen W, Welfle K, et al (2004) Recognition of DNA by ω protein from the broad-host range *Streptococcus pyogenes* plasmid pSM19035: analysis of binding to operator DNA with one to four heptad repeats. Nucl Acids Res 32: 3136–3147. 15190131 10.1093/nar/gkh633 PMC434439

[32] Welfle K, Pratto F, Misselwitz R, Behlke J, Alonso JC, Welfle K (2005) Role of the N-terminal region and of β-sheet residue Thr29 on the activity of the ω_2_ global regulator from the broad-host range *Streptococcus pyogenes* plasmid pSM19035. Biol Chem 386: 881–894. 16164413 10.1515/BC.2005.103

[33] Ceglowski P, Boitsov A, Karamyan N, Chai S, Alonso JC (1993) Characterization of the effectors required for stable inheritance of *Streptococcus pyogenes* pSM19035-derived plasmids in *Bacillus subtilis* . Mol Gen Genet 241: 579–585. 8264532 10.1007/BF00279900

[34] Cardenas PP, Carrasco B, Sanchez H, Deikus G, Bechhofer DH, Alonso JC (2009) *Bacillus subtilis* polynucleotide phosphorylase 3'→5' DNase activity is involved in DNA repair. Nucl Acids Res 37: 4157–4169. 10.1093/nar/gkp314 19433509 PMC2709576

[35] Carrasco B, Ayora S, Lurz R, Alonso JC (2005) *Bacillus subtilis* RecU Holliday-junction resolvase modulates RecA activities. Nucl Acids Res 33: 3942–3952. 16024744 10.1093/nar/gki713 PMC1176016

[36] Wang JD, Rokop ME, Barker MM, Hanson NR, Grossman AD (2004) Multicopy plasmids affect replisome positioning in *Bacillus subtilis* . J Bacteriol 186: 7084–7090. 15489419 10.1128/JB.186.21.7084-7090.2004 PMC523195

[37] Lemon KP, Grossman AD (2000) Movement of replicating DNA through a stationary replisome. Mol Cell 6: 1321–1330. 11163206 10.1016/s1097-2765(00)00130-1

[38] Schofield WB, Lim HC, Jacobs-Wagner C (2010) Cell cycle coordination and regulation of bacterial chromosome segregation dynamics by polarly localized proteins. EMBO J 29: 3068–3081. 10.1038/emboj.2010.207 20802464 PMC2944072

[39] Ah-Seng Y, Rech J, Lane D, Bouet JY (2013) Defining the role of ATP hydrolysis in mitotic segregation of bacterial plasmids. PLoS Genet 9: e1003956 10.1371/journal.pgen.1003956 24367270 PMC3868542

[40] Vecchiarelli AG, Mizuuchi K, Funnell BE (2012) Surfing biological surfaces: exploiting the nucleoid for partition and transport in bacteria. Mol Microbiol 86: 513–523. 10.1111/mmi.12017 22934804 PMC3481007

[41] Szardenings F, Guymer D, Gerdes K (2011) ParA ATPases can move and position DNA and subcellular structures. Curr Op Microbiol 14: 712–718. 10.1016/j.mib.2011.09.008 21963112

[42] Ebersbach G, Ringgaard S, Moller-Jensen J, Wang Q, Sherratt DJ, Gerdes K (2006) Regular cellular distribution of plasmids by oscillating and filament-forming ParA ATPase of plasmid pB171. Mol Microbiol 61: 1428–1442. 16899080 10.1111/j.1365-2958.2006.05322.x

