# Supplementary figures and images for: ParAB Partition Dynamics in Firmicutes: Nucleoid Bound ParA Captures and Tethers ParB-Plasmid Complexes
Source: PLoS One. 2015 Jul 10;10(7):e0131943. doi: 10.1371/journal.pone.0131943 (PMC4498918; doi:10.1371/journal.pone.0131943)

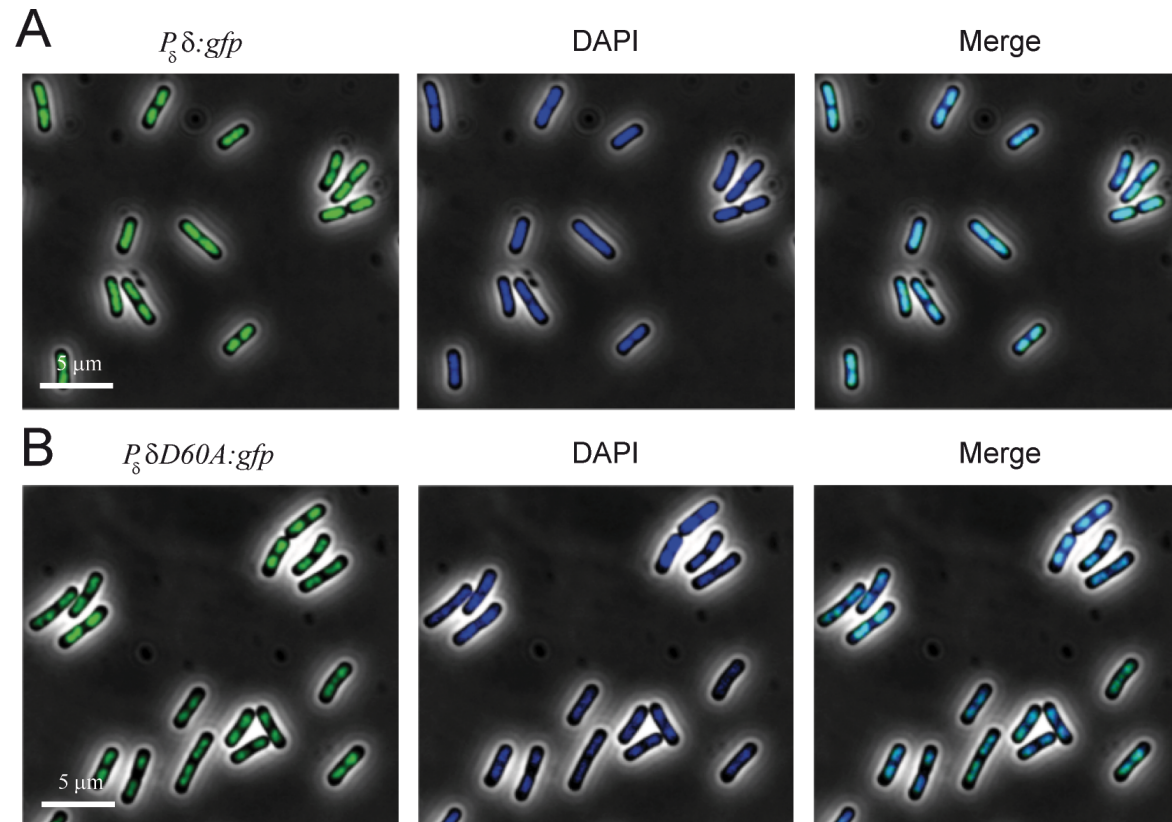

**S1 Fig. Subcellular localization of  $(\delta:GFP)_2$  or  $(\delta D60A:GFP)_2$  in the absence of  $\omega_2$**

Supplement: S1 Fig — Cells bearing plasmid-borne P δ δ:gfp (A) or P δ δD60A·gfp gene (B) were grown in MMS7 at 30°C. Fluorescence images of cells, images of the same cells stained with DAPI to show DNA, and the merge of both images are shown. Scale bar is 5 μm. (PDF) [file pone.0131943.s001.pdf]

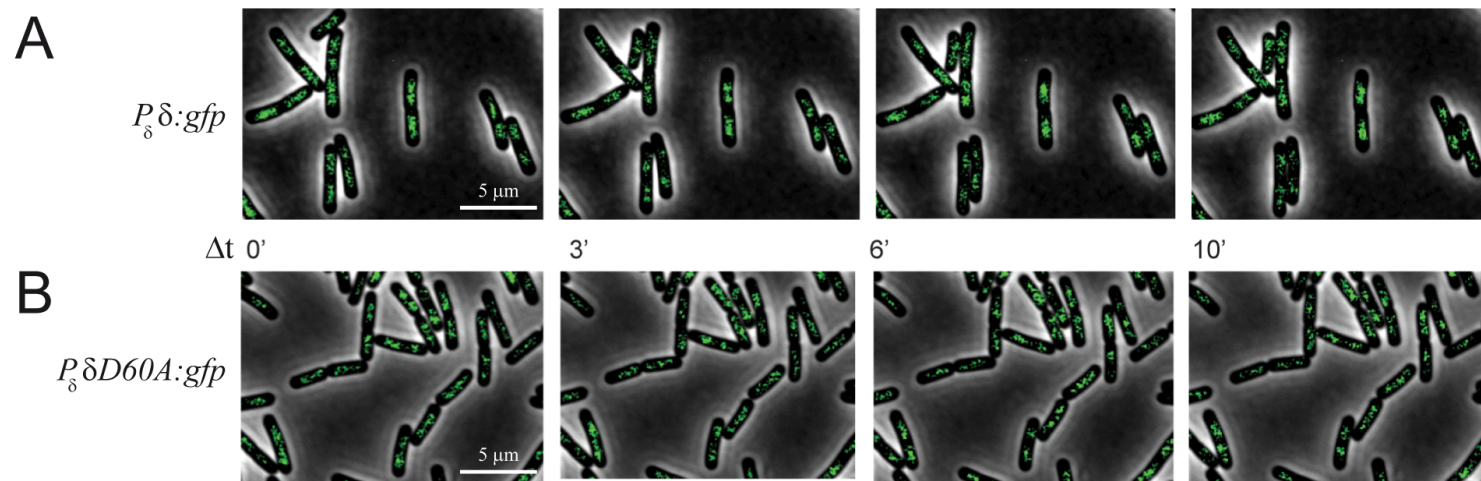

**S2 Fig. Time lapse of  $(\delta:GFP)_2$  or  $(\delta D60A:GFP)_2$  fluorescence**

Supplement: S2 Fig — Cells bearing-plasmid borne P δ δ:gfp (A) or P δ δD60A:gfp gene (B) were grown in MMS7 at 30°C. Images of the same cells with fluorescence from (δ·GFP)2 or (δD60A·GFP)2 are shown for the indicated time. Scale bar is 5 μm. (PDF) [file pone.0131943.s002.pdf]

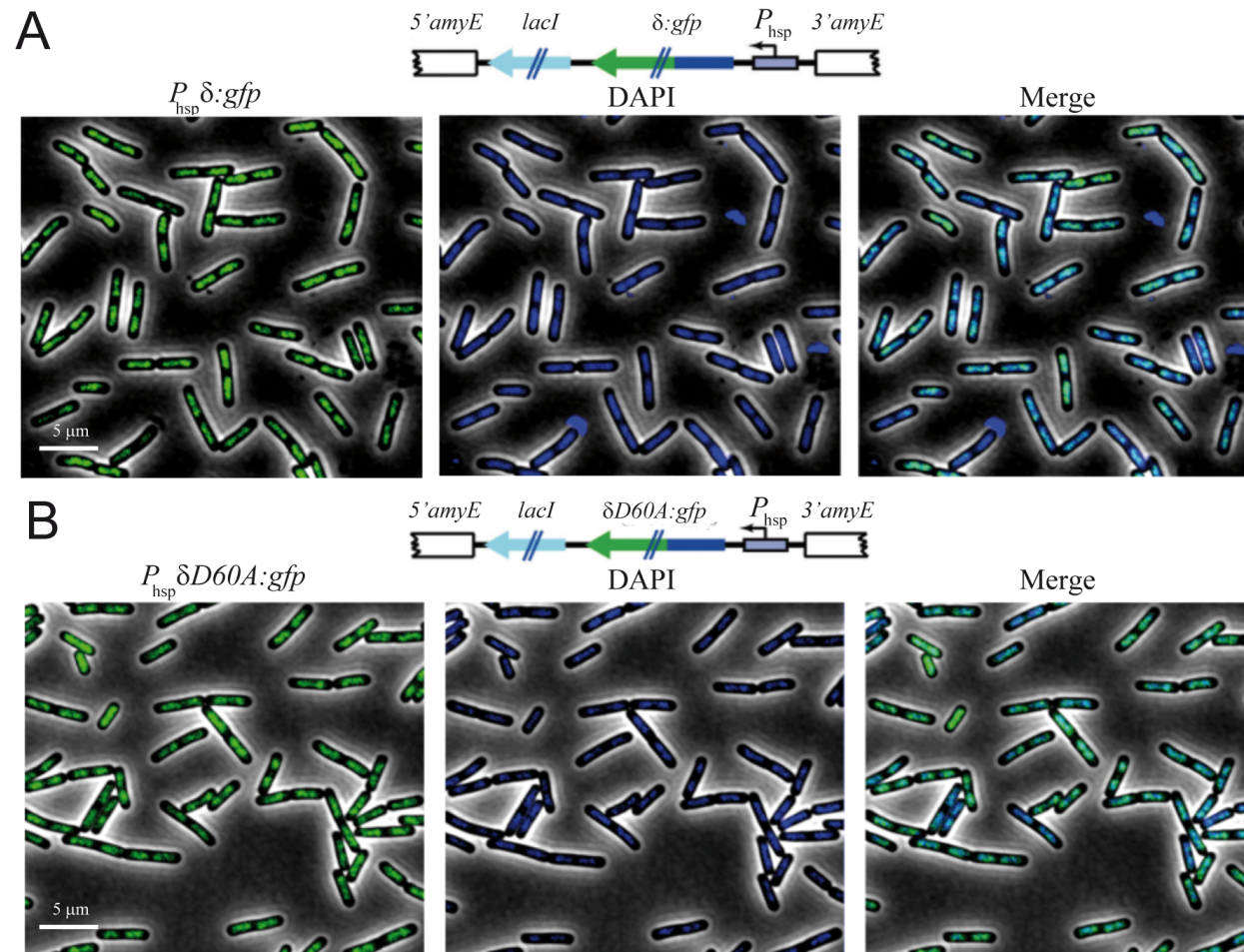

**S3 Fig. Subcellular localization of  $(\delta:GFP)_2$  or  $(\delta D60A:GFP)_2$**

Supplement: S3 Fig — Illustration showing the structure of the P hsp δ:gfp (A) or P hsp δD60A:gfp (B) expression cassettes integrated in the host chromosome, rendering strains BG947 and BG1097, respectively. Cells were grown in MMS7 at 30°C in the presence of 10 μM IPTG. GFP fluorescence images of cells, images of the same cells stained with DAPI to show DNA, and the merge of both images are shown. Scale bar is 5 μm. (PDF) [file pone.0131943.s003.pdf]
