# Supplementary material for: ParAB Partition Dynamics in Firmicutes: Nucleoid Bound ParA Captures and Tethers ParB-Plasmid Complexes
Source: PLoS One. 2015 Jul 10;10(7):e0131943. doi: 10.1371/journal.pone.0131943 (PMC4498918; doi:10.1371/journal.pone.0131943)
Supplement: S1 Table — (DOCX) [file pone.0131943.s004.docx]

**S1 Table**. **Strains and plasmids**

| *E. coli* strain | Genotype/relevant properties | Source |
| --- | --- | --- |
| ER2560 | *fhuA*2 *lacZ*::T7 *gene*1 [*lon*] ompT *gal* R(*mcr*-73::miniTn10-Tet^S^) endA1 *sulA*11 [dcm] R(*zgb*-210::Tn10Tet^S^) Δ(*mcrC*-*mrr*)114::IS10. | New England Biolabs |
| XL1-Blue | *recA*1 *endA*1 *gyrA*96 *thi-*1 *hsdR*17 *supE*44 *relA*1 *lac*[F ́ *proAB* *lacI*^q^ Z∆M15 Tn10 (Tet^R^)]. | Stratagene |
| *E. coli*  Plasmid | Plasmid-borne gene(s), site(s) | Source |
| pCB30 | pUC18-borne *parS*2 (*P*_ω_) | [[1](#_ENREF_1)] |
| pT712 | expression vector | GIBCO-BRL |
| pT712ω | pT712-borne ω | [[2](#_ENREF_2)] |
| pCB746 | pT712-borne *δ* | [[3](#_ENREF_3)] |
| pCB755 | pT712-borne *δD60A* | [[3](#_ENREF_3)] |
| pCB853 | pT712-borne δ*K242A* | [[4](#_ENREF_4)] |
| pCB855 | pT712-borne δ*D211A* | [[4](#_ENREF_4)] |
| pCB872 | pT712-borne δ*D60A D211A* | This work |
| pDR111 | *Amy*-front, hyper-spank promoter (*P_hsp_*), *lac*I, *amy*-back | [[5](#_ENREF_5)] |
| *B. subtilis*  Strain | Genotype/relevant properties | Source |
| BG214 | *trpCE metA*5 *amyE1 rsbV*37 *xre*1 *xkd*A1 *att*^SPß^ *att*^ICE^*^Bs^*^1^ | Our collection |
| BG947 | + IPTG inducible *amyE*:*P_hsp_* δ:*gfp* gene, *spc* | [[3](#_ENREF_3)] |
| BG1097 | + IPTG inducible *amyE*:*P_hsp_* δ*D60A*:*gfp* gene, *spc* | [[3](#_ENREF_3)] |
| BG1469 | + IPTG inducible *amyE*:*P_hsp_* ω:*yfp* gene, *spc* | This work |
| BG1447 | + IPTG inducible *amyE*:*P_hsp_* ωΔN19:*yfp* gene, *spc* | This work |
| BG508 | + *amyE*:*P*_δ_ *lacZ*, *cat* | [[1](#_ENREF_1)] |
| BG1311 | + *amyE*:*P*_xylE_ *lacI*-*gfp*, *cat* | This work |
| *B. subtilis*  Plasmid^a^ | Plasmid-borne gene(s), site(s) | Source |
| pHP14 | shuttle plasmid, *cat*, *ery* | [[2](#_ENREF_2)] |
| pHP14ω | *parS2* (*P*_ω_) site ω | [[2](#_ENREF_2)] |
| pBT291 | *parS1* (*P*_δ_) site δ | [[6](#_ENREF_6)] |
| pBT346 | *parS* sites, *P*_δ_ δ and *P*_ω_ ω | [[6](#_ENREF_6)] |
| pCB578 | *parS1* (*P*_δ_) site, δ:*gfp* | [[3](#_ENREF_3)] |
| pCB586 | *parS2* (*P*_ω_) site, ω | [[3](#_ENREF_3)] |
| pCB706 | *parS* sites, *P*_δ_ δ and *P*_ω_ ω | [[3](#_ENREF_3)] |
| pCB702 | *parS* sites, *P*_δ_ *δ*:*gfp* and *P*_ω_ ω | [[3](#_ENREF_3)] |
| pCB703 | *parS* sites, *P*_δ_ δ and *P*_ω_ ωΔN19 | [[3](#_ENREF_3)] |
| pCB760 | *parS1* (*P*_δ_) site, δD60A:*gfp* | [[3](#_ENREF_3)] |
| pCB761 | *parS* sites, *P*_δ_ δD60A:*gfp* and *P*_ω_ ω | [[3](#_ENREF_3)] |
| pCB742 | *parS2* (*P*_ω_) site, ωΔN19 | [[3](#_ENREF_3)] |
| pCB840 | *parS* sites, *P*_δ_ δD60A:*gfp,* *P*_ω_ ωΔN19 | [[3](#_ENREF_3)] |
| pCB945 | *parS* sites, *P*_δ_ δ and *P*_ω_ ω, *lacO* | This work |
| pCB841 | *parS* sites, *P*_δ_ δ:*cfp*, *P*_ω_ ω:*yfp* | This work |
| pCB846 | *parS2* (*P*_ω_) site, ω:*yfp* | This work |
| pCB843 | *parS* sites, *P*_δ_ δ, *P*_ω_ ω:*yfp* | This work |
| pCB873 | *parS* sites, *P*_δ_ δK242A:*gfp* | This work |
| pCB874 | *parS* sites, *P*_δ_ δD211A:*gfp* | This work |
| pCB861 | *parS* sites, *P*_δ_ δK242A:*gfp* and *P*_ω_ ω | This work |
| pCB860 | *parS* sites, *P*_δ_ δD211A:g*fp* and *P*_ω_ ω | This work |
| pCB881 | *parS* sites, *P*_δ_ δD60 D211A and *P*_ω_ ω | This work |

^a^Mid-copy number (8 ± 1 copies/cell) pHP14 or derivatives bearing the δ and ω genes (or their variants) under the controls of their own promoters [*P*_δ_ (*parS*1) and *P*_ω_ (*parS*2) see Figure 1].

References

1. de la Hoz AB, Ayora S, Sitkiewicz I, Fernandez S, Pankiewicz R, et al. (2000) Plasmid copy-number control and better-than-random segregation genes of pSM19035 share a common regulator. Proc Natl Acad Sci U S A 97: 728-733.

2. Welfle K, Pratto F, Misselwitz R, Behlke J, Alonso JC, et al. (2005) Role of the N-terminal region and of β-sheet residue Thr29 on the activity of the ω_2_ global regulator from the broad-host range *Streptococcus pyogenes* plasmid pSM19035. Biol Chem 386: 881-894.

3. Pratto F, Cicek A, Weihofen WA, Lurz R, Saenger W, et al. (2008) *Streptococcus pyogenes* pSM19035 requires dynamic assembly of ATP-bound ParA and ParB on *parS* DNA during plasmid segregation. Nucleic Acids Res 36: 3676-3689.

4. Soberón NE, Lioy VS, Pratto F, Volante A, Alonso JC (2011) Molecular anatomy of the *Streptococcus pyogenes* pSM19035 partition and segrosome complexes. Nucleic Acids Res 39: 2624-2637.

5. Graham TG, Wang X, Song D, Etson CM, van Oijen AM, et al. (2014) ParB spreading requires DNA bridging. Genes Dev 28: 1228-1238.

6. Ceglowski P, Alonso JC (1994) Gene organization of the *Streptococcus pyogenes* plasmid pDB101: sequence analysis of the orf η-*copS* region. Gene 145: 33-39.
