# Supplementary material for: ParAB Partition Dynamics in Firmicutes: Nucleoid Bound ParA Captures and Tethers ParB-Plasmid Complexes
Source: PLoS One. 2015 Jul 10;10(7):e0131943. doi: 10.1371/journal.pone.0131943 (PMC4498918; doi:10.1371/journal.pone.0131943)
Supplement: S2 Table — (DOCX) [file pone.0131943.s005.docx]

**S2 Table.** **Relative binding of δ_2_ or its variants to nsDNA**

| Protein | Nucleotide cofactor | (K_Dapp_) in nM^a^ |
| --- | --- | --- |
| δ_2_ | ATP | ~ 140 |
| δ_2_D60A | ATP | ~ 75 |
| δ_2_D211A | ATP | ~ 25 |
| δ_2_D60A D211A | ATP | ~ 6 |
| δ_2_K242A | ATP | ~ 3000 |
| δ_2_ | - | > 2000 |
| δ_2_ | ADP | > 2000 |
| δ_2_D60A | ADP | > 2000 |
| δ_2_D211A | ADP | > 2000 |
| δ_2_D60A D211A | ADP | > 2000 |
| δ_2_K242A | ADP | > 2000 |

A [α^32^P]-labelled-423-bp long DNA (0.1 nM) segment was incubated with increasing concentrations of the indicated protein for 15 min at 37°C in buffer B containing or not 1 mM ATP or ADP, then the K_Dapp_ was determined by EMSA. Samples were separated by 6% PAGE, and the formation of protein-DNA complexes was quantified as described in Materials and Methods. The K_Dapp_ values (in nM) are the average of at least three independent experiments and are within a 10% standard error.

References

1. de la Hoz AB, Ayora S, Sitkiewicz I, Fernandez S, Pankiewicz R, et al. (2000) Plasmid copy-number control and better-than-random segregation genes of pSM19035 share a common regulator. Proc Natl Acad Sci U S A 97: 728-733.

2. Welfle K, Pratto F, Misselwitz R, Behlke J, Alonso JC, et al. (2005) Role of the N-terminal region and of β-sheet residue Thr29 on the activity of the ω_2_ global regulator from the broad-host range *Streptococcus pyogenes* plasmid pSM19035. Biol Chem 386: 881-894.

3. Pratto F, Cicek A, Weihofen WA, Lurz R, Saenger W, et al. (2008) *Streptococcus pyogenes* pSM19035 requires dynamic assembly of ATP-bound ParA and ParB on *parS* DNA during plasmid segregation. Nucleic Acids Res 36: 3676-3689.

4. Soberón NE, Lioy VS, Pratto F, Volante A, Alonso JC (2011) Molecular anatomy of the *Streptococcus pyogenes* pSM19035 partition and segrosome complexes. Nucleic Acids Res 39: 2624-2637.

5. Graham TG, Wang X, Song D, Etson CM, van Oijen AM, et al. (2014) ParB spreading requires DNA bridging. Genes Dev 28: 1228-1238.

6. Ceglowski P, Alonso JC (1994) Gene organization of the *Streptococcus pyogenes* plasmid pDB101: sequence analysis of the orf η-*copS* region. Gene 145: 33-39.
