# Supplementary material for: ParAB Partition Dynamics in Firmicutes: Nucleoid Bound ParA Captures and Tethers ParB-Plasmid Complexes
Source: PLoS One. 2015 Jul 10;10(7):e0131943. doi: 10.1371/journal.pone.0131943 (PMC4498918; doi:10.1371/journal.pone.0131943)
Supplement: S3 Table — (DOCX) [file pone.0131943.s006.docx]

**S3 Table.** **Protein (ω:YFP)_2_ binds *P*_δ_ and represses transcription**

| Gene(s) *in trans*^a^ | β-Galactosidase activity^b^  *P*_δ_ (*parS*1) *lac*Z |
| --- | --- |
| - | 1845 ± 120 |
| *P*_ω_ ω | 19.5 ± 1.7 |
| *P*_ω_ ω, *P*_δ_ δ | 14.3 ± 1.2 |
| *P*_ω_ ωΔN19 | 38.3 ± 3.3 |
| *P*_ω_ ωΔN19, *P*_δ_ δ | 39.1 ± 4.4 |
| *P*_ω_ ω:*yfp* | 48.8 ± 3.7 |
| *P*_ω_ ω:*yfp*, *P*_δ_ δ | 20.6 ± 1.6 |
| *P*_ω_ *yfp*:ω | 1882 ± 127 |
| *P*_ω_ *yfp*:ω, *P*_δ_ δ | 1824 ± 144 |

^a^Plasmid-borne genes transcribed from their native promoters and expressed *in trans*. ^b^Expression of β-galactosidase activity in *B. subtilis* cells bearing the *P*_δ_ *lacZ* gene in the presence of various products *in trans*. The activity is expressed in Miller units as described in Materials and Methods.

References

1. de la Hoz AB, Ayora S, Sitkiewicz I, Fernandez S, Pankiewicz R, et al. (2000) Plasmid copy-number control and better-than-random segregation genes of pSM19035 share a common regulator. Proc Natl Acad Sci U S A 97: 728-733.

2. Welfle K, Pratto F, Misselwitz R, Behlke J, Alonso JC, et al. (2005) Role of the N-terminal region and of β-sheet residue Thr29 on the activity of the ω_2_ global regulator from the broad-host range *Streptococcus pyogenes* plasmid pSM19035. Biol Chem 386: 881-894.

3. Pratto F, Cicek A, Weihofen WA, Lurz R, Saenger W, et al. (2008) *Streptococcus pyogenes* pSM19035 requires dynamic assembly of ATP-bound ParA and ParB on *parS* DNA during plasmid segregation. Nucleic Acids Res 36: 3676-3689.

4. Soberón NE, Lioy VS, Pratto F, Volante A, Alonso JC (2011) Molecular anatomy of the *Streptococcus pyogenes* pSM19035 partition and segrosome complexes. Nucleic Acids Res 39: 2624-2637.

5. Graham TG, Wang X, Song D, Etson CM, van Oijen AM, et al. (2014) ParB spreading requires DNA bridging. Genes Dev 28: 1228-1238.

6. Ceglowski P, Alonso JC (1994) Gene organization of the *Streptococcus pyogenes* plasmid pDB101: sequence analysis of the orf η-*copS* region. Gene 145: 33-39.
